# Acoustic stability of a self-gravitating cylinder leading to astrostructure formation

**DOI:** 10.1038/s41598-023-34415-1

**Published:** 2023-05-03

**Authors:** Sayanti Dasgupta, Ahmed Atteya, Pralay Kumar Karmakar

**Affiliations:** 1 Department of Physics, Tezpur University, Napaam, 784028 Tezpur, Assam India; 2 Department of Physics, Faculty of Science, Alexandria University, P.O. 21511, Alexandria, Egypt

**Keywords:** Astronomy and planetary science, Physics

## Abstract

We employ a quantum hydrodynamic model to investigate the cylindrical acoustic waves excitable in a gyromagnetoactive self-gravitating viscous cylinder comprised of two-component (electron–ion) plasma. The electronic equation of state incorporates the effect of temperature degeneracy. It reveals an expression for the generalized pressure capable of reproducing a completely degenerate (CD) quantum (Fermi) pressure and a completely non-degenerate (CND) classical (thermal) pressure. A standard cylindrical wave analysis, moderated by the Hankel function, yields a generalized linear (sextic) dispersion relation. The low-frequency analysis is carried out procedurally in four distinct parametric special cases of astronomical importance. It includes the quantum (CD) non-planar (cylindrical), quantum (CD) planar, classical (CND) non-planar (cylindrical), and classical (CND) planar. We examine the multi-parametric influences on the instability dynamics, such as the plasma equilibrium concentration, kinematic viscosity, and so forth. It is found that, in the quantum regime, the concentration plays a major role in the system destabilization. In the classical regime, the plasma temperature plays an important role in both the stabilization and destabilization. It is further seen that the embedded magnetic field influences the instability growth dynamics in different multiparametric regimes extensively, and so forth. The presented analysis can hopefully be applicable to understand the cylindrical acoustic wave dynamics leading actively to the formation of astrophysical gyromagnetic (filamentary) structures in diverse astronomical circumstances in both the classical and quantum regimes of astronomical relevance.

## Introduction

The study of acoustic waves and instabilities excitable in two-component plasmas (electron–ion) has recently gathered significant research interest because of their large-scale applications in diversified explorative areas in both the classical and quantum regimes. Such plasmas are naturalistically ubiquitous in diverse circumstances. It mainly includes inertially confined laboratory plasmas, liquid metals, stellar and planetary interiors, Earth’s auroral regions, Jupiter magnetosphere, supernova explosions, and so on^1–6^. As a consequence, it is quite expedient to analyze the supported normal acoustic waves and instabilities in order to perceive their bulk stability behaviours in different astronomical circumstances.

It is worth mentioning in the above context that a good number of rigorous investigations have been performed to study the dynamics of acoustic waves and corresponding instabilities in astrophysical plasmas^7–14^. In this context, an investigative study of non-linear electron acoustic waves in quantum plasmas has been performed^7^. It has been found that both compressive and rarefactive solitons along with periodical potential structures exist for various ranges of dimensionless quantum parameter^7^. In addition, linear and non-linear quantum ion-acoustic waves in dense magnetized electron–positron-ion plasmas have also been analyzed^8^. It has been found that the ion-acoustic soliton structures are influenced by several factors, like quantum pressure, concentration of positrons, and so on^8^. Non-linear quantum dust acoustic waves in non-uniform complex dusty plasma have also been studied^9^. It has been found that the system admits only rarefactive solitons, the properties of which were analyzed using the initial ion and electron number densities^9^. A theoretic study has been performed to analyze the obliquely propagating two-dimensional quantum dust ion-acoustic solitary waves in magnetized quantum dusty plasma by deriving the Zakharov-Kuznetsov (ZK) equation for small-amplitude perturbations^10^. The combined effects of obliqueness and non-extensive electrons have also been incorporated in the study of the ion-acoustic waves to investigate the propagation properties of two possible modes in the linear regime^11^. It has been found that electron non-extensivity decreases the phase velocity of both the modes^11^. It has also been observed that the relativistic effect plays an important role in the propagation of the positron-acoustic solitary waves^12^. The linear and non-linear analyses of dust-acoustic waves in dissipative space dusty plasma have also been addressed^13^. The dependence of the damping rate of the waves on the wavenumber, kinematic viscosity, and so on has been discussed^13^. The acoustic mode excitation and stability in strongly coupled bi-component plasma have also been investigated^14^. It has been found that the acoustic mode is significantly modified due to consideration of the viscoelastic effect^14^. Another semi-analytic study has been conducted to analyze the effects of positron density and temperature on the electron acoustic shock waves in magnetized electron–positron-ion plasma^15^. It has been found that combined action of dissipation, superthermality, concentration of positrons; and so on significantly modify the properties of the electron-acoustic shock waves^15^. The formation of electron acoustic solitary structure in the inner magnetosphere of Earth has also been studied^16^. The non-linear coupling between electromagnetic waves and electron-acoustic waves in astrophysical plasma has also been investigated^17^. From this study, it has been found that the high-frequency electromagnetic waves interact non-linearly with the electron-acoustic waves^17^. The propagation dynamics of non-linear electron-acoustic waves have been explored with the help of the Boussinesq equation^18^. It has been found that electron-acoustic waves possess breather structures, in addition to the solitary wave solutions^18^. A semi-analytic theoretic study has also been conducted to study the dynamics of the nucleus-acoustic waves, excitable in compact astroobjects in spherical geometry^19^. The influence of relativistic effects, electrostatic confinement pressure, and other realistic factors on the propagation of ion-acoustic waves has been investigated^20^. The instability arising due to the interaction of the electromagnetic waves with the small-frequency longitudinal spin electron-acoustic waves has been investigated^21^. Very recently, a review article has also been compiled taking into account all the possible acoustic modes excitable in Jovian dusty magnetosphere^22^. The characteristics of dust acoustic cnoidal waves due to dust particle polarization have been investigated^23^. It can be fairly concluded that acoustic waves have attracted the attention of researchers since a long time. It is clearly evident that even though there are quite a large number of investigations dealing with acoustic waves in cylindrical and spherical geometry, the cylindrical acoustic wave analysis by employing Hankel function is missing from all the aforementioned investigations to the best of our knowledge. By cylindrical waves, we mean a wave where distribution of all quantities is homogeneous in some direction and has complete axial symmetry about that direction^24^. Thus, investigation of the characteristics of cylindrical acoustic waves by means of the Hankel function formalism in a uniformly rotating magnetized plasma system in planar and non-planar regimes is still an open problem hitherto lying unexplored.

In the present semi-analytic investigation, we consider a generalized quantum two-fluid hydrodynamic model consisting of electrons and singly charged ions. The two-component plasma is confined in a magnetized axisymmetric cylinder rotating uniformly about the longitudinal direction. The electrons and ions are governed by their appropriate equations of state. The fermions governed by the Fermi–Dirac statistical distribution law are characterized by temperature $\left( T \right)$ and chemical potential $\left( \mu \right)$^25–27^. The effect of temperature degeneracy considered here is incorporated in the electronic equation of state with the help of the temperature degeneracy parameter given as $G^{\prime}_{e} = {{Li_{{{5 \mathord{\left/ {\vphantom {5 2}} \right. \kern-0pt} 2}}} \,\left( { - \xi } \right)} \mathord{\left/ {\vphantom {{Li_{{{5 \mathord{\left/ {\vphantom {5 2}} \right. \kern-0pt} 2}}} \,\left( { - \xi } \right)} {Li_{{{3 \mathord{\left/ {\vphantom {3 2}} \right. \kern-0pt} 2}}} }}} \right. \kern-0pt} {Li_{{{3 \mathord{\left/ {\vphantom {3 2}} \right. \kern-0pt} 2}}} }}\,\left( { - \xi } \right)$, where $\xi \left( {\mu ,T} \right) = e^{\beta \mu }$ and $\beta = {1 \mathord{\left/ {\vphantom {1 {k_{B} T}}} \right. \kern-0pt} {k_{B} T}}$^25–27^. Under application of appropriate approximations, the electronic equation of state results in the completely degenerate (CD) quantum pressure (Fermi) and the completely non-degenerate (CND) classical pressure (thermal). The ionic equation of state takes into account the classical thermal pressure. A standard normal cylindrical wave analysis by employing the Hankel function^24^ yields a sextic dispersion relation, which is then analyzed in the low-frequency (LF) regime. The modified dispersion relation is then investigated in the light of four different parametric windows. The influence of various realistic parameters like equilibrium number density, kinematic viscosity, and so forth on the instability dynamics is thoroughly studied. The importance of cylindrical geometry considered here can be justified from the fact that axisymmetric cylinders under self-gravity offer insights on evolution of elongated molecular cloud, magnetized arms of spiral galaxies, circumnuclear starburst rings and filamentary structures of various scales in broad astrophysical and cosmological contexts^28–30^.

## Physical model formalism

We consider a magnetized axisymmetric cylindrical two-component plasma system subjected to the non-local self-gravitational action. It consists of electrons and singly charged ions. The former is judiciously modelled with the help of generalized quantum hydrodynamic formalism; whereas, the latter is treated classically. This model evolves under the conjoint influence of the Lorentz force, Coriolis rotation, kinematic viscosity, Bohm potential, and temperature degeneracy effects. The confining cylinder rotates with a constant angular velocity directed along the longitudinal direction. The basic governing equations here consist of continuity equation, force-balancing momentum equation, and appropriate equation of state. The system closure is obtained with the help of electrostatic and gravitational Poisson equations. The quantum dynamics of the electronic species in generic notations is accordingly cast as

1 $$\partial_{t} n_{e} + \left( r \right)^{ - 1} \partial_{r} \left( {rn_{e} u{}_{e}} \right) = 0,$$

2 $$\partial_{t} u_{e} = \left( {em_{e}^{ - 1} } \right)\partial_{r} \varphi_{E} - eB_{z} m_{e}^{ - 1} u_{e\varphi } + \left( {m_{e} n_{e}^{ - 1} } \right)\partial_{r} P_{e} + \hbar^{2} \left( {2m_{e}^{2} } \right)^{ - 1} \partial_{r} n_{e}^{{ - {1 \mathord{\left/ {\vphantom {1 2}} \right. \kern-0pt} 2}}} \left\{ {r^{ - 1} \partial_{r} \left( {rn_{e}^{{{1 \mathord{\left/ {\vphantom {1 2}} \right. \kern-0pt} 2}}} } \right)} \right\} + 2v_{\varphi } \omega_{z} - \partial_{r} \psi ,$$

3 $$P_{e} = \,\,\,G_{e}^{\prime } n_{e} \beta^{ - 1} .$$

Likewise, the classical dynamics of the ionic species in our considered plasma is described as

4 $$\partial_{t} n_{i} + \left( r \right)^{ - 1} \partial_{r} \left( {rn_{i} u{}_{i}} \right) = 0,$$

5 $$\partial_{t} u_{i} = \left( {em_{i}^{ - 1} } \right)\partial_{r} \varphi_{E} - eB_{z} m_{i}^{ - 1} u_{i\varphi } + \left( {m_{i} n_{i} } \right)^{ - 1} \partial_{r} P_{i} + 2v_{\varphi } \omega_{z} + \left( {m_{i} n_{i} } \right)^{ - 1} \eta \left( r \right)^{ - 1} \partial_{r} \left( {r\partial_{r} u_{i} } \right) - \partial_{r} \psi ,$$

6 $$P_{i} = \,n_{i} k_{B} T.$$

The model is systematically closed with the help of electrostatic and self-gravitational Poisson equations given respectively in customary notations as

7 $$\left( r \right)^{ - 1} \partial_{r} \left( {r\partial_{r} \varphi_{E} } \right) = e\left( {\varepsilon_{0}^{ - 1} } \right)\left( {n_{e} - n_{i} } \right),$$

8 $$\left( r \right)^{ - 1} \partial_{r} \left( {r\partial_{r} \psi } \right) = 4\pi G\left( {\Delta \rho_{e} + \Delta \rho_{i} } \right).$$

In our considered cylindrical coordinate system, $r$ and *t* denote the spatial and temporal parameters, respectively. $n_{e\left( i \right)}$ and $u_{e\left( i \right)}$, denote the population density and the velocity of the electrons (ions) with their inertial mass $m_{e\left( i \right)}$, respectively. $\omega_{ge(i)} = {{eB_{z} } \mathord{\left/ {\vphantom {{eB_{z} } {m_{e(i)} }}} \right. \kern-0pt} {m_{e(i)} }}$ is the electronic (ionic) magnetic gyrofrequency, where $B_{z}$ is the magnetic field acting along the longitudinal direction. The axisymmetric plasma system is assumed to be rotating with a constant angular velocity $\omega$. The constant rotational force acting on the entire system is given by $C_{F}^{*} = 2v_{\varphi } \omega_{z}$, where $\omega_{z}$ is the longitudinal component of the angular velocity and $v_{\varphi }$ is the azimuthal component of linear velocity. $P_{e\left( i \right)}$ gives the pressure acting on the electronic (ionic) species. $h = 6.6 \times 10^{ - 34}$ J s is the Planck constant signifying the unit of quantum–mechanical action. $T$ is the thermal temperature on the Kelvin scale. $k_{B} = 1.38 \times 10^{ - 23}$ J K^-1^ is the Boltzmann constant representing the energy-temperature coupling. $\varphi_{E}$ and $\psi$ are the electrostatic potential and gravitational potential, respectively. $\varepsilon_{0} = 8.85 \times 10^{ - 12}$ F m^-1^ is the permittivity of free space (here, the plasma). $G = 6.67 \times 10^{ - 11}$ N m^2^ kg^-2^ is the universal gravitational constant, also called the Newtonian constant, signifying the coupling strength of gravitating matter. In Eq. (8), $\Delta \rho_{e(i)} = \left( {\rho_{e(i)} - \rho_{0} } \right) = m_{e(i)} \left( {n_{e(i)} - n_{0} } \right)$ is the effective plasma matter density used to model the so-called Jeans swindle, extensively adopted as an ad-hoc self-gravitational homogenization technique without any loss of generality of the fluctuation dynamics under consideration.

A number of physical points regarding the above mathematical equations are noteworthy. Here, Eq. (1) is the equation of continuity, depicting the flux conservation of the electronic fluid. Then, Eq. (2) is the force balancing (momentum) equation. Here, the force by virtue of electronic motion (L.H.S) is balanced by the forces arising due to electrostatic potential (1st term in R.H.S), magnetic field (2nd term in R.H.S), electronic pressure (3rd term in R.H.S), quantum Bohm potential (4th term in R.H.S), Coriolis rotation (5th term in R.H.S), and gravitational potential (6th term in R.H.S). The electronic equation of state incorporating the temperature degeneracy effects is represented by Eq. (3). The arbitrary temperature degeneracy in usual notations^25–27^ is given as $G^{\prime}_{e} = {{Li_{{{5 \mathord{\left/ {\vphantom {5 2}} \right. \kern-0pt} 2}}} \,\left( { - \xi } \right)} \mathord{\left/ {\vphantom {{Li_{{{5 \mathord{\left/ {\vphantom {5 2}} \right. \kern-0pt} 2}}} \,\left( { - \xi } \right)} {Li_{{{3 \mathord{\left/ {\vphantom {3 2}} \right. \kern-0pt} 2}}} }}} \right. \kern-0pt} {Li_{{{3 \mathord{\left/ {\vphantom {3 2}} \right. \kern-0pt} 2}}} }}\,\left( { - \xi } \right)$. Here, $Li_{p} \left( { - \xi } \right)$ is the polylogarithmic function with index *p*. $\xi \left( {\mu ,T} \right) = e^{\beta \mu } = e^{{{\mu \mathord{\left/ {\vphantom {\mu {k_{B} T}}} \right. \kern-0pt} {k_{B} T}}}}$ describes the degeneracy of the system^25–27^. The general form of $Li_{p} \left( { - \xi } \right)$ for $p\, > 0$ is given as

9 $$Li_{p} \left( { - \xi } \right)\, = - \left( {\Gamma \left( p \right)} \right)^{ - 1} \int\limits_{0}^{\infty } {t^{p - 1} \left( {e^{t} \xi^{ - 1} + 1} \right)^{ - 1} } dt\,\,;$$

where, $\Gamma \left( p \right) = \int\limits_{0}^{\infty } {x^{p - 1} } e^{ - x} \,dx$ is the gamma function with an argument *p*. For the CD limit ($\xi \to \infty$), one arrives at

10 $$G^{\prime}_{e} = 2\left( {5\delta } \right)^{ - 1} ,$$

11 $$P_{e} = \,\left( {3\pi^{2} } \right)^{{{2 \mathord{\left/ {\vphantom {2 3}} \right. \kern-0pt} 3}}} \hbar^{2} n_{e}^{{{5 \mathord{\left/ {\vphantom {5 3}} \right. \kern-0pt} 3}}} \left( {5m_{e} } \right)^{ - 1} ;$$

where, $\delta = {T \mathord{\left/ {\vphantom {T {T_{F} }}} \right. \kern-0pt} {T_{F} }}$ denotes the ratio between the thermal and Fermi temperature; and Eq. (11) gives the CD electronic pressure (quantum).

It is noteworthy that, in the presence of a uniform magnetic field (*B*) in the z-direction, we have the transverse pressure, $P_{ \bot }$ and parallel pressure, $P_{||}$. It is noteworthy that $P_{ \bot }$ and $P_{||}$ become significantly different for large values of *B*. For $B \to \infty$, only the lowest Landau level contributes to $P_{||}$ and $P_{ \bot }$ becomes zero in the considered limit^31^. The quantization of the Landau levels is also important in the quantum scenarios. Thus, the different kinds of magnetic pressures in a quantum system^31^ are related to the quantization of the Landau levels. But, the effect of the Landau levels can only be observed when the mean thermal energy (classical) is smaller than the energy level separation (quantum), that is, $k_{B} T < < \hbar \omega$. In the proposed work, $k_{B} T = 1.38 \times 10^{ - 20}$ J for $T = 10^{3}$ K, and $\hbar \omega_{e} = \hbar \left( {{{eB} \mathord{\left/ {\vphantom {{eB} {m_{e} }}} \right. \kern-0pt} {m_{e} }}} \right) = 1.84 \times 10^{ - 22}$ J for $B = 10$ T. This implies that $k_{B} T > > \hbar \omega$. Thus, the Landau levels and related magnetic pressure effects have been ignored in the considered study without any loss of generality in a justified way.

For the CND limit ($\xi \to 0$), one gets

12 $$G^{\prime}_{e} = 1,$$

13 $$P_{e} = \,n_{e} k_{B} T.$$

Equation (13) gives the classical electronic pressure for the CND limit.

It is evident that Eq. (4) is the ionic analog of Eq. (1). Likewise, Eq. (5) is the exact analog of Eq. (2), except the Bohm potential term, since the ions are treated classically because of their large mass. The ionic fluid is characterized with the kinematic viscosity (5th term in R.H.S), in addition to all the forces already mentioned before in the case of Eq. (3). Now, Eq. (6) is the ionic equation of state modelled classically. The model closure is obtained with the help of electrostatic and gravitational Poisson equations (Eqs. (7) and (8)).

For a scale-invariant analysis, we adopt a standard normalization scheme^20^, quite relevant for astrophysical description. The corresponding normalized sets of equations are cast as

14 $$\partial_{\tau } N_{e} + \left( R \right)^{ - 1} \partial_{R} \left( {RN_{e} M{}_{e}} \right) = 0,$$

15.1 $$\begin{aligned} N_{e} \partial_{\tau } M_{e} = & \left( {m_{i} m_{e}^{ - 1} } \right)N_{e} \partial_{R} \Phi_{E} - N_{e} \Omega_{ge}^{*} M_{e\varphi } + 3^{ - 1} M_{Fe}^{2} \partial_{R} N_{e} \\ & + 4^{ - 1} H_{p}^{2} \left\{ {\partial_{R}^{3} N_{e} + \left( {R^{ - 1} } \right)\partial_{R}^{2} N_{e} - \left( {R^{ - 2} } \right)\partial_{R} N_{e} } \right\} \\ & + 2N_{e} M_{\varphi } \omega_{z}^{*} - N_{e} \partial_{R} \Psi ,\,\,\,\,\,\,\,\,\,\,\,\,\,\,\,\,\left( {{\text{for}}\,{\text{CD}}\,{\text{case}}} \right){,} \\ \end{aligned}$$

15.2 $$\begin{aligned} N_{e} \partial_{\tau } M_{e} = & \left( {m_{i} m_{e}^{ - 1} } \right)N_{e} \partial_{R} \Phi_{E} - N_{e} \Omega_{ge}^{*} M_{e\varphi } + \left( {m_{i} m_{e}^{ - 1} } \right)\partial_{R} \left( {N_{e} T^{*} } \right) \\ & + 2N_{e} M_{\varphi } \omega_{z}^{*} - N_{e} \partial_{R} \Psi ,\,\,\,\,\,\,\,\,\,\,\,\,\,\,\,\,\,\,\,\,\,\,\,\,\left( {{\text{for}}\,{\text{CND}}\,{\text{case}}} \right){,} \\ \end{aligned}$$

16 $$\partial_{\tau } N_{i} + \left( R \right)^{ - 1} \partial_{R} \left( {RN_{i} M{}_{i}} \right) = 0,$$

17 $$N_{i} \partial_{\tau } M_{i} = - N_{i} \partial_{R} \Phi_{E} + N_{i} \Omega_{gi}^{*} M_{i\varphi } + \partial_{R} \left( {N_{i} T^{*} } \right) + 2N_{i} M_{\varphi } \omega_{z}^{*} + \eta^{*} \left( R \right)^{ - 1} \partial_{R} \left( {R\partial_{R} M_{i} } \right) - N_{i} \partial_{R} \Psi ,$$

18 $$\left( R \right)^{ - 1} \partial_{R} \left( {R\partial_{R} \Phi_{E} } \right) = \left( {N_{e} - N_{i} } \right),$$

19 $$\left( R \right)^{ - 1} \partial_{R} \left( {R\partial_{R} \Psi } \right) = \sigma \left\{ {m_{e} m_{i}^{ - 1} \left( {N_{e} - 1} \right) + \left( {N_{i} - 1} \right)} \right\}.$$

In the above, the spatial coordinate $r$ is normalized as $R\, = \,{r \mathord{\left/ {\vphantom {r {L_{0} }}} \right. \kern-0pt} {L_{0} }}\,$; where, $L_{0} \, = \,\,{{c_{s} } \mathord{\left/ {\vphantom {{c_{s} } {\omega_{pi} }}} \right. \kern-0pt} {\omega_{pi} }}\,\,\,$ is a characteristic spatial scale. $c_{s} = \sqrt {{{2E_{Fe} } \mathord{\left/ {\vphantom {{2E_{Fe} } {m_{i} }}} \right. \kern-0pt} {m_{i} }}} = {{hn_{0} } \mathord{\left/ {\vphantom {{hn_{0} } {4\sqrt {m_{e} m_{i} } }}} \right. \kern-0pt} {4\sqrt {m_{e} m_{i} } }}$ is the acoustic speed in terms of Fermi energy. $\omega_{pi} \, = \,\sqrt {{{n_{0} e^{2} } \mathord{\left/ {\vphantom {{n_{0} e^{2} } {\varepsilon_{0} m_{i} }}} \right. \kern-0pt} {\varepsilon_{0} m_{i} }}}$ designates the ion plasma oscillation frequency. The temporal coordinate $t$ is normalized as $\tau \, = \,{t \mathord{\left/ {\vphantom {t {\omega_{pi}^{ - 1} }}} \right. \kern-0pt} {\omega_{pi}^{ - 1} }}\,\,\,$. The Fermi energy is given as $E_{Fe} \, = \,\,{{p_{F}^{2} } \mathord{\left/ {\vphantom {{p_{F}^{2} } {2\,m_{e} }}} \right. \kern-0pt} {2\,m_{e} }}\,$, with $p_{F} \, = \,{{h\,n_{0} } \mathord{\left/ {\vphantom {{h\,n_{0} } 4}} \right. \kern-0pt} 4}$ as the corresponding Fermi momentum. The rescaled electronic (ionic) number density is given as $N_{e\left( i \right)\,} \, = \,{{n_{e\left( i \right)\,} } \mathord{\left/ {\vphantom {{n_{e\left( i \right)\,} } {n_{0} }}} \right. \kern-0pt} {n_{0} }}$, where $n_{0}$ is the equilibrium number density. $M_{e\left( i \right)} \, = \,{{u_{e\,\left( i \right)} } \mathord{\left/ {\vphantom {{u_{e\,\left( i \right)} } {c_{s} }}} \right. \kern-0pt} {c_{s} }}$ gives the Mach number of the electronic (ionic) species. The normalized electronic (ionic) magnetic gyrofrequency is given as $\Omega_{ge(i)}^{*} \, = \,{{\omega_{ge(i)} } \mathord{\left/ {\vphantom {{\omega_{ge(i)} } {\omega_{pi} }}} \right. \kern-0pt} {\omega_{pi} }}\,\,\,$. The normalized Coriolis rotational force is given as $C_{F}^{*} = M_{\varphi } \omega_{z}^{*}$; where, $M_{\varphi } = {{v_{\varphi } } \mathord{\left/ {\vphantom {{v_{\varphi } } {c_{s} }}} \right. \kern-0pt} {c_{s} }}$ is the rescaled tangential velocity of the system and $\omega_{z}^{*} = {{\omega_{z} } \mathord{\left/ {\vphantom {{\omega_{z} } {\omega_{pi} }}} \right. \kern-0pt} {\omega_{pi} }}$ is the rescaled longitudinal component of angular velocity. $M_{Fe} = {{v_{Fe} } \mathord{\left/ {\vphantom {{v_{Fe} } {c_{s} }}} \right. \kern-0pt} {c_{s} }}$ is the Fermi Mach number, where, $v_{Fe}$ is the Fermi velocity. $H_{p} = {{\hbar \omega_{pi} } \mathord{\left/ {\vphantom {{\hbar \omega_{pi} } {m_{e} c_{s}^{2} }}} \right. \kern-0pt} {m_{e} c_{s}^{2} }}$ is the quantum parameter. $\sigma = {{\omega_{Ji}^{2} } \mathord{\left/ {\vphantom {{\omega_{Ji}^{2} } {\omega_{pi}^{2} }}} \right. \kern-0pt} {\omega_{pi}^{2} }}$ gives the ratio of the squares of Jeans frequency to that of ionic plasma oscillation frequency. $\omega_{Ji} = \sqrt {4\pi Gm_{i} n_{0} }$ gives the Jeans frequency for ions. The normalized kinematic viscosity is given as $\eta^{*} = {\eta \mathord{\left/ {\vphantom {\eta {m_{i} n_{0} c_{s} L_{0} }}} \right. \kern-0pt} {m_{i} n_{0} c_{s} L_{0} }}$. $T^{*} = {{Tk_{B} } \mathord{\left/ {\vphantom {{Tk_{B} } {m_{i} c_{s}^{2} }}} \right. \kern-0pt} {m_{i} c_{s}^{2} }}$ gives the normalized temperature. In a similar pattern, $\Phi_{E} = {{e\varphi_{E} } \mathord{\left/ {\vphantom {{e\varphi_{E} } {2E_{Fe} }}} \right. \kern-0pt} {2E_{Fe} }}$ is the normalized electrostatic potential. The normalized gravitational potential is given as $\Psi = {\psi \mathord{\left/ {\vphantom {\psi {c_{s}^{2} }}} \right. \kern-0pt} {c_{s}^{2} }}$.

It is to be noted that in the quantum regime, Bohm potential term accounts for the typical quantum like behaviour like tunneling, overlapping of wave packets, and so on. Thus in the CND (classical) regime represented by Eq. (15.2), normalized Bohm potential term is ignored^32,33^.

## Perturbation analysis

We linearly perturb the relevant physical fluid parameters appearing in Eqs. (14), (15.1), (15.2), (16), (17), (18) and (19), using a cylindrical wave analysis^24^ in an autonormalized Fourier transformed wavespace given as

20 $$F\left( {R,\,\tau } \right) = F_{0} + \,F_{1} \left( {R,\,\tau } \right)\, = F_{0} + F_{10} \exp \left( { - i\Omega \tau } \right)H_{0}^{\left( 1 \right)} \left( {k^{*} R} \right)\,\,,$$

where $H_{0}^{\left( 1 \right)}$ is the Hankel function of the first kind, of order 0.

For $R \to 0$, $H_{0}^{\left( 1 \right)}$ has logarithmic singularity:

21 $$H_{0}^{\left( 1 \right)} = \left( {2i\pi^{ - 1} } \right)\log \left( {k^{*} R} \right)$$

At large distances, we have

22 $$H_{0}^{\left( 1 \right)} = \left( {2\pi^{ - 1} } \right)^{{{1 \mathord{\left/ {\vphantom {1 2}} \right. \kern-0pt} 2}}} \left( {k^{*} R} \right)^{{ - {1 \mathord{\left/ {\vphantom {1 2}} \right. \kern-0pt} 2}}} \exp \left[ {i\left( {k^{*} R - \pi 4^{ - 1} } \right)} \right]$$

Thus, Eq. (20) gets modified as

23 $$F\left( {R,\,\tau } \right) = F_{0} + \,F_{1} \left( {R,\,\tau } \right)\, = F_{0} + F_{10} \left( {2\pi^{ - 1} } \right)^{{{1 \mathord{\left/ {\vphantom {1 2}} \right. \kern-0pt} 2}}} \left( {k^{*} R} \right)^{{ - {1 \mathord{\left/ {\vphantom {1 2}} \right. \kern-0pt} 2}}} \exp \left[ {i\left( {k^{*} R - \Omega \tau - \pi 4^{ - 1} } \right)} \right]\,,$$

24 $$F = \left[ {N_{s} \,\,\,\,\,\,\,\,M_{s} \,\,\,\,\,\,\,\,\Phi_{E} \,\,\,\,\,\,\,\,\,\,\Psi } \right]^{T} ,$$

25 $$F_{0} = \left[ {1\,\,\,\,\,\,\,\,\,\,\,0\,\,\,\,\,\,\,\,\,\,\,\,\,\,0\,\,\,\,\,\,\,\,\,\,\,\,0\,} \right]^{T} \,,$$

26 $$F_{1} = \left[ {N_{s1} \,\,\,\,M_{s1} \,\,\,\,\,\,\Phi_{E1} \,\,\,\,\,\,\,\Psi_{1} \,} \right]^{T} .$$

Here, we assume an axisymmetric cylinder such that all quantities are homogeneously distributed along z-direction, and thereby just show radial variations. In Eq. (23), *F*_*1*_ denotes the radial perturbations, which evolve as per the Hankel function of first kind of order 0. *F*_*0*_ denotes the equilibrium values corresponding to which perturbations *F*_*1*_ take place. In the new Fourier transformed wavespace, the spatial and temporal operators get transformed as ${\partial \mathord{\left/ {\vphantom {\partial {\partial R \to \left( {\,ik^{*} - \,\,{1 \mathord{\left/ {\vphantom {1 R}} \right. \kern-0pt} R}} \right)}}} \right. \kern-0pt} {\partial R \to \left( {\,ik^{*} - \,\,{1 \mathord{\left/ {\vphantom {1 R}} \right. \kern-0pt} R}} \right)}}$ and ${\partial \mathord{\left/ {\vphantom {\partial {\partial \tau \to \left( { - i\,\,\Omega } \right)}}} \right. \kern-0pt} {\partial \tau \to \left( { - i\,\,\Omega } \right)}}$, respectively. Here, $\Omega$ $\left( {{{ = \omega } \mathord{\left/ {\vphantom {{ = \omega } {\omega_{pi} }}} \right. \kern-0pt} {\omega_{pi} }}} \right)$ denotes the normalized fluctuation frequency and $k^{*} \left( {\sim {k \mathord{\left/ {\vphantom {k {L_{0}^{ - 1} }}} \right. \kern-0pt} {L_{0}^{ - 1} }}} \right)$ designates the normalized wavenumber. The linearly perturbed relevant physical parameters from Eqs. (14), (15.1), (15.2), (16), (17), (18) and (19) in the new wave space can respectively be cast as

27 $$N_{e1} = - i\Omega^{ - 1} \left\{ {ik^{*} + \,\left( {2R} \right)^{ - 1} \,} \right\}M_{e1} ,$$

28 $$M_{e1} = E^{ - 1} \left( {m_{i} m_{e}^{ - 1} } \right)\left\{ {ik^{*} - \,\left( {2R} \right)^{ - 1} \,} \right\}\Phi_{E1} - iE^{ - 1} \Omega^{ - 1} \sigma \left\{ {k^{{*^{2} }} + \left( {4R^{2} } \right)^{ - 1} } \right\}\left\{ { - k^{{*^{2} }} + \left( {4R^{2} } \right)^{ - 1} } \right\}^{ - 1} M_{i1} ,$$

29 $$N_{i1} = - i\Omega^{ - 1} \left\{ {ik^{*} + \,\left( {2R} \right)^{ - 1} \,} \right\}M_{i1} ,$$

30 $$M_{i1} = - H^{ - 1} \left\{ {ik^{*} - \,\left( {2R} \right)^{ - 1} \,} \right\}\Phi_{E1} \left[ {1 + i\Omega^{ - 1} E^{ - 1} \sigma \left\{ {k^{{*^{2} }} + \left( {4R^{2} } \right)^{ - 1} } \right\}\left\{ { - k^{{*^{2} }} + \left( {4R^{2} } \right)^{ - 1} } \right\}^{ - 1} } \right],$$

31 $$\Phi_{E1} = - i\Omega^{ - 1} \left\{ {ik^{*} + \,\left( {2R} \right)^{ - 1} \,} \right\}\left\{ { - k^{{*^{2} }} + \left( {4R^{2} } \right)^{ - 1} } \right\}^{ - 1} \left( {M_{e1} - M_{i1} } \right),$$

32 $$\Psi_{1} = - i\Omega^{ - 1} \sigma \left\{ {ik^{*} + \,\left( {2R} \right)^{ - 1} \,} \right\}\left\{ { - k^{{*^{2} }} + \left( {4R^{2} } \right)^{ - 1} } \right\}^{ - 1} \left\{ {\left( {m_{e} m_{i}^{ - 1} } \right)M_{e1} + M_{i1} } \right\}.$$

In the above set of Eqs. (27), (28), (29), (30), (31) and (32), the various substituted terms are given as

33 $$E = - i\Omega + i\Omega^{ - 1} \left\{ {ik^{*} + \,\left( {2R} \right)^{ - 1} \,} \right\}\left. {\left[ {{ - }\Omega_{ge}^{*} M_{e\varphi } + 4^{ - 1} H_{p}^{2} B_{p} + 2M_{\varphi } \omega_{z}^{*} + } \right.\left\{ {ik^{*} - \,\left( {2R} \right)^{ - 1} \,} \right\}\left[ {\alpha - \left\{ { - k^{{*^{2} }} + \left( {4R^{2} } \right)^{ - 1} } \right\}^{ - 1} \sigma m_{e} m_{i}^{ - 1} } \right]} \right]$$

34 $$B_{p} = - ik^{{*^{3} }} + k^{{*^{2} }} \left( {2R} \right)^{ - 1} + ik^{*} \left( {4R^{2} } \right)^{ - 1} - 5\left( {8R^{3} } \right)^{ - 1}$$

35 $$\alpha = \left( 3 \right)^{ - 1} M_{Fe}^{2} ,\,{\text{for CDcase}},{\text{ where}}\,M_{Fe} = {{v_{Fe} } \mathord{\left/ {\vphantom {{v_{Fe} } {c_{s} }}} \right. \kern-0pt} {c_{s} }}\,{\text{and}}\,v_{Fe} = \left( {3\pi^{2} n_{e} } \right)^{{{1 \mathord{\left/ {\vphantom {1 3}} \right. \kern-0pt} 3}}} \hbar \,m_{e}^{ - 1}$$

36 $$\alpha = m_{i} m_{e}^{ - 1} T^{*} ,\,{\text{for CND}}\,{\text{case, }}$$

37 $$\begin{aligned} H = & - i\Omega + i\Omega^{ - 1} \left\{ {ik^{*} + \,\left( {2R} \right)^{ - 1} \,} \right\}\left. {\left[ {\Omega_{gi}^{*} M_{i\varphi } + 2M_{\varphi } \omega_{z}^{*} + } \right.\left\{ {ik^{*} - \,\left( {2R} \right)^{ - 1} \,} \right\}\left[ {T^{*} - \sigma \left\{ { - k^{{*^{2} }} + \left( {4R^{2} } \right)^{ - 1} } \right\}^{ - 1} } \right]} \right] \\ & - \left\{ { - k^{{*^{2} }} + \left( {4R^{2} } \right)^{ - 1} } \right\}\eta^{*} + \Omega^{ - 2} \left\{ {k^{{*^{2} }} + \left( {4R^{2} } \right)^{ - 1} } \right\}^{2} \left\{ { - k^{{*^{2} }} + \left( {4R^{2} } \right)^{ - 1} } \right\}^{ - 2} \sigma^{2} m_{e} m_{i}^{ - 1} E^{ - 1} \\ \end{aligned}$$

After a standard procedure of elimination and substitution among Eqs. (27), (28), (29), (30), (31), (32), (33), (34), (35), (36) and (37), we obtain a generalized linear sextic dispersion relation cast as

38 $$\Omega^{6} + A_{5} \Omega^{5} + A_{4} \Omega^{4} + A_{3} \Omega^{3} + A_{2} \Omega^{2} + A_{1} \Omega + A_{0} = 0$$

The different coefficients in an expanded form are given as

39 $$A_{5} = - i\left\{ {k^{{*^{2} }} + \left( {4R^{2} } \right)^{ - 1} } \right\}\,\eta^{*}$$

$$A_{4} = \left\{ {ik^{*} + \,\left( {2R} \right)^{ - 1} \,} \right\}\,\,\left[ { - 2\Omega_{ge}^{*} M_{e\varphi }^{*} + \left( {2\alpha + T^{*} } \right)} \right.\left\{ {ik^{*} - \,\left( {2R} \right)^{ - 1} \,} \right\} - 2^{ - 1} H_{p}^{2} B_{p} + 6M_{\varphi } \omega_{z}^{*} + \Omega_{gi}^{*} M_{i\varphi }^{*}$$

40 $$\left. { + \left\{ {ik^{*} - \,\left( {2R} \right)^{ - 1} \,} \right\}\left\{ { - k^{{*^{2} }} + \left( {4R^{2} } \right)^{ - 1} } \right\}^{ - 1} \left\{ {m_{i} m_{e}^{ - 1} + 1 - \sigma \left( {2m_{e} m_{i}^{ - 1} + 1} \right)} \right\}} \right]$$

$$A_{3} = - i\left\{ {ik^{*} + \,\left( {2R} \right)^{ - 1} \,} \right\}\eta^{*} \left[ {\left\{ { - k^{{*^{2} }} + \left( {4R^{2} } \right)^{ - 1} } \right\}\,\,\,\left[ {2\Omega_{ge}^{*} M_{e\varphi }^{*} + 2^{ - 1} H_{p}^{2} B_{p} - 4M_{\varphi } \omega_{z}^{*} - 2\alpha \left\{ {ik^{*} - \,\left( {2R} \right)^{ - 1} \,} \right\}} \right]} \right.$$

41 $$\left. { + \left\{ {ik^{*} - \,\left( {2R} \right)^{ - 1} \,} \right\}\left( {2\sigma m_{e} m_{i}^{ - 1} - m_{i} m_{e}^{ - 1} } \right)} \right],$$

$$A_{2} = - \left\{ {ik^{*} + \,\left( {2R} \right)^{ - 1} \,} \right\}^{2} \left[ {\,\left\{ {ik^{*} - \,\left( {2R} \right)^{ - 1} \,} \right\}\,\left[ { - 2\alpha \,\left( {\Omega_{ge}^{*} M_{e\varphi }^{*} - \Omega_{gi}^{*} M_{i\varphi }^{*} } \right) + 2^{ - 1} H_{p}^{2} B_{p} \alpha + 4M_{\varphi } \omega_{z}^{*} \left( {\alpha + T^{*} } \right)} \right.\,\,\,\,\,\,\,\,\,\,\,\,\,\,\,\,\,\,\,\,\,\,\,\,\,\,\,\,\,} \right.$$

$$- 2\left( {\Omega_{ge}^{*} M_{e\varphi }^{*} T^{*} - 2M_{\varphi } \omega_{z}^{*} \alpha } \right) - 2^{ - 1} T^{*} H_{p}^{2} B_{p} + \left\{ { - k^{{*^{2} }} + \left( {4R^{2} } \right)^{ - 1} } \right\}^{ - 1} \left[ {2\Omega_{ge}^{*} M_{e\varphi } \sigma \left( {1 + m_{e} m_{i}^{ - 1} } \right)} \right.$$

$$- 2^{ - 1} H_{p}^{2} B_{p} \sigma \left( { - 1 + m_{e} m_{i}^{ - 1} } \right) - 2\sigma \left\{ {\left( {\Omega_{gi}^{*} M_{i\varphi } + 4M_{\varphi } \omega_{z}^{*} } \right)m_{e} m_{i}^{ - 1} + 2M_{\varphi } \omega_{z}^{*} } \right\} + 2\left\{ {\Omega_{ge}^{*} M_{e\varphi } + 2M_{\varphi } \omega_{z}^{*} } \right.$$

$$\left. {\left. {\left. { - 4^{ - 1} H_{p}^{2} B_{p} } \right\} + m_{i} m_{e}^{ - 1} \left( {\Omega_{gi}^{*} M_{i\varphi } + 4M_{\varphi } \omega_{z}^{*} - \Omega_{ge}^{*} M_{e\varphi } + 4^{ - 1} H_{p}^{2} B_{p} } \right)} \right]\,\,} \right]\, + \left\{ {ik^{*} - \,\left( {2R} \right)^{ - 1} \,} \right\}^{2} \left[ {\alpha^{2} } \right.$$

$$+ 2\alpha T^{*} + \left\{ { - k^{{*^{2} }} + \left( {4R^{2} } \right)^{ - 1} } \right\}^{ - 1} \left[ { - 2\sigma \left\{ {\left( {T^{*} + 1} \right)m_{e} m_{i}^{ - 1} + 1} \right\}} \right. + 2\alpha + m_{i} m_{e}^{ - 1} \left. {\left( {T^{*} + \alpha } \right)} \right] + \left\{ { - k^{{*^{2} }} + \left( {4R^{2} } \right)^{ - 1} } \right\}^{ - 2}$$

$$\left. {\left[ { - \sigma \left( {1 + m_{i} m_{e}^{ - 1} } \right)} \right. - 2\sigma m_{e} m_{i}^{ - 1} + \sigma^{2} m_{e} m_{i}^{ - 1} \left. {\left( {1 + m_{e} m_{i}^{ - 1} } \right) + 2\sigma } \right]\,\,} \right] - 2^{ - 1} H_{p}^{2} B_{p} \left( {\Omega_{ge}^{*} M_{e\varphi }^{*} + \Omega_{gi}^{*} M_{i\varphi }^{*} } \right)$$

42 $$\left. { - 8M_{\varphi } \omega_{z}^{*} \Omega_{ge}^{*} M_{e\varphi } + \left( {\Omega_{ge}^{*} M_{e\varphi }^{*} } \right)^{2} + \left( {4^{ - 1} H_{p}^{2} B_{p} } \right)^{2} + 2\Omega_{gi}^{*} M_{i\varphi } \left( { - \Omega_{ge}^{*} M_{e\varphi }^{*} + 2M_{\varphi } \omega_{z}^{*} } \right) + 12\left( {M_{\varphi } \omega_{z}^{*} } \right)^{2} } \right],$$

$$A_{1} = - i\left\{ {ik^{*} + \,\left( {2R} \right)^{ - 1} \,} \right\}^{2} \eta^{*} \,\left[ {\,\left[ {\left\{ {ik^{*} - \,\left( {2R} \right)^{ - 1} \,} \right\}\left[ {\left( {\Omega_{ge}^{*} M_{e\varphi } - 4^{ - 1} H_{p}^{2} B_{p} - 2M_{\varphi } \omega_{z}^{*} } \right)\left[ {2\sigma m_{e} m_{i}^{ - 1} - m_{i} m_{e}^{ - 1} } \right.} \right.} \right.} \right.$$

$$\left. {\left. { - 2\alpha \left\{ { - k^{{*^{2} }} + \left( {4R^{2} } \right)^{ - 1} } \right\}} \right]\,\,} \right]\, + \left\{ { - k^{{*^{2} }} + \left( {4R^{2} } \right)^{ - 1} } \right\}\,\,\left[ {\Omega_{ge}^{*} M_{e\varphi } \left( {\Omega_{ge}^{*} M_{e\varphi } - 2^{ - 1} H_{p}^{2} B_{p} - 4M_{\varphi } \omega_{z}^{*} } \right)} \right.$$

$$\left. {\left. { + 4^{ - 1} H_{p}^{2} B_{p} \left( {4^{ - 1} H_{p}^{2} B_{p} + 4M_{\varphi } \omega_{z}^{*} } \right) + 4\left( {M_{\varphi } \omega_{z}^{*} } \right)^{2} } \right]\,\,} \right] + \left\{ {ik^{*} - \,\left( {2R} \right)^{ - 1} \,} \right\}^{2} \left[ { - 2\alpha \sigma \,m_{e} m_{i}^{ - 1} } \right. + \alpha \,m_{i} m_{e}^{ - 1}$$

43 $$\left. {\left. { + \left\{ { - k^{{*^{2} }} + \left( {4R^{2} } \right)^{ - 1} } \right\}^{ - 1} \left( {\sigma \,m_{e} m_{i}^{ - 1} - m_{i} m_{e}^{ - 1} } \right) + 2\alpha^{2} \left\{ { - k^{{*^{2} }} + \left( {4R^{2} } \right)^{ - 1} } \right\}} \right]\,\,} \right]$$

$$A_{0} = \left[ { - P + \left[ {\left\{ {k^{{*^{2} }} + \left( {4R^{2} } \right)^{ - 1} } \right\}\left\{ { - k^{{*^{2} }} + \left( {4R^{2} } \right)^{ - 1} } \right\}^{ - 1} m_{i} m_{e}^{ - 1} Q} \right] + \left[ {2\left\{ {k^{{*^{2} }} + \left( {4R^{2} } \right)^{ - 1} } \right\}^{2} \left\{ { - k^{{*^{2} }} + \left( {4R^{2} } \right)^{ - 1} } \right\}^{ - 2} \sigma S} \right]} \right.$$

44 $$\left. { - \left[ {\left\{ {k^{{*^{2} }} + \left( {4R^{2} } \right)^{ - 1} } \right\}^{3} \left\{ { - k^{{*^{2} }} + \left( {4R^{2} } \right)^{ - 1} } \right\}^{ - 2} \sigma^{2} } \right] + \left[ {\left\{ {k^{{*^{2} }} + \left( {4R^{2} } \right)^{ - 1} } \right\}\left\{ { - k^{{*^{2} }} + \left( {4R^{2} } \right)^{ - 1} } \right\}^{ - 1} I} \right]} \right]$$

The different terms substituted in *A*_*0*_ are given in an expanded form as

$$P = - \left\{ {ik^{*} + \,\left( {2R} \right)^{ - 1} \,} \right\}^{3} \left[ {\left\{ {ik^{*} - \,\left( {2R} \right)^{ - 1} \,} \right\}\left[ {\,\left[ {2\alpha \Omega_{gi}^{*} M_{i\varphi } \left( {\Omega_{ge}^{*} M_{e\varphi } - 2M_{\varphi } \omega_{z}^{*} } \right)} \right.} \right.} \right. - 4^{ - 1} H_{p}^{2} B_{p} \left\{ {2\alpha \Omega_{gi}^{*} M_{i\varphi } } \right. + 4\left. {\left( {T^{*} + \alpha } \right)M_{\varphi } \omega_{z}^{*} } \right\}$$

$$- \Omega_{ge}^{*} M_{e\varphi } T^{*} \left( {\Omega_{ge}^{*} M_{e\varphi } - 4M_{\varphi } \omega_{z}^{*} } \right) - 4^{ - 1} H_{p}^{2} B_{p} T^{*} \left\{ { - 2\Omega_{ge}^{*} M_{e\varphi } } \right. + \left. {4^{ - 1} H_{p}^{2} B_{p} } \right\} - 4M_{\varphi } \omega_{z}^{*} \left\{ {T^{*} } \right.M_{\varphi } \omega_{z}^{*}$$

$$\left. {\left. { + \left( {2M_{\varphi } \omega_{z}^{*} - \Omega_{ge}^{*} M_{e\varphi } } \right)\alpha } \right\}} \right]\, + \left\{ { - k^{{*^{2} }} + \left( {4R^{2} } \right)^{ - 1} } \right\}^{ - 1} \left[ { - 2\Omega_{gi}^{*} M_{i\varphi } \sigma \,m_{e} m_{i}^{ - 1} \left( {\Omega_{ge}^{*} M_{e\varphi } - 4^{ - 1} H_{p}^{2} B_{p} - 4M_{\varphi } \omega_{z}^{*} } \right)} \right.$$

$$- 4\sigma m_{e} m_{i}^{ - 1} M_{\varphi } \omega_{z}^{*} \left( {\Omega_{ge}^{*} M_{e\varphi } - 4^{ - 1} H_{p}^{2} B_{p} - 2M_{\varphi } \omega_{z}^{*} } \right) + \sigma 4^{ - 1} H_{p}^{2} B_{p} \left( {4^{ - 1} H_{p}^{2} B_{p} + 4M_{\varphi } \omega_{z}^{*} } \right)$$

$$\left. {\left. { - 2\Omega_{ge}^{*} M_{e\varphi } \sigma \left\{ {2M_{\varphi } \omega_{z}^{*} \left( {1 - M_{\varphi } \omega_{z}^{*} } \right) + 4^{ - 1} H_{p}^{2} B_{p} - 2^{ - 1} \Omega_{ge}^{*} M_{e\varphi } } \right\}} \right]\,} \right] + \left\{ {ik^{*} - \,\left( {2R} \right)^{ - 1} \,} \right\}^{2} \left[ {\left[ { - \alpha \left( {\alpha \Omega_{gi}^{*} M_{i\varphi } } \right.} \right.} \right.$$

$$\left. {\left. { - 2\Omega_{ge}^{*} M_{e\varphi } T^{*} + \,\,2\alpha M_{\varphi } \omega_{z}^{*} \,} \right) - 2\alpha T^{*} \left( {2M_{\varphi } \omega_{z}^{*} + 4^{ - 1} H_{p}^{2} B_{p} } \right)} \right] + \left\{ { - k^{{*^{2} }} + \left( {4R^{2} } \right)^{ - 1} } \right\}^{ - 1} \left[ {\sigma m_{e} m_{i}^{ - 1} } \right.\left\{ {\Omega_{gi}^{*} M_{i\varphi } \alpha \,} \right.$$

$$\left. {\left. { + T^{*} \left( {2M_{\varphi } \omega_{z}^{*} - \Omega_{ge}^{*} M_{e\varphi } } \right.} \right) + m_{i} m_{e}^{ - 1} \alpha 4^{ - 1} H_{p}^{2} B_{p} } \right\} + \sigma m_{e} m_{i}^{ - 1} \left\{ {2^{ - 1} H_{p}^{2} B_{p} T + 4\alpha M_{\varphi } \omega_{z}^{*} + 2} \right.m_{i} m_{e}^{ - 1} \left( {2\alpha } \right.M_{\varphi } \omega_{z}^{*}$$

$$\left. {\left. {\left. { - \Omega_{ge}^{*} M_{e\varphi } } \right)} \right\}} \right] + \left\{ { - k^{{*^{2} }} + \left( {4R^{2} } \right)^{ - 1} } \right\}^{ - 2} \left[ { - \sigma^{2} \left( {m_{e} m_{i}^{ - 1} } \right)^{2} } \right.\left( {\Omega_{{{\text{gi}}}}^{*} M_{i\varphi } - 2m_{i} m_{e}^{ - 1} \Omega_{{{\text{ge}}}}^{*} M_{e\varphi } + 2M_{\varphi } \omega_{z}^{*} } \right) + \sigma^{2} m_{e} m_{i}^{ - 1}$$

$$\left. {\left. {\left( {2^{ - 1} M_{\varphi } \omega_{z}^{*} + \Omega_{ge}^{*} M_{e\varphi } \, + 4^{ - 1} H_{p}^{2} B_{p} } \right)} \right]\,} \right]\,\, + \left\{ {ik^{*} - \,\left( {2R} \right)^{ - 1} \,} \right\}^{3} \left[ { - \alpha^{2} } \right.T^{*} + \left\{ { - k^{{*^{2} }} + \left( {4R^{2} } \right)^{ - 1} } \right\}^{ - 1} \left( {2\alpha \sigma m_{e} m_{i}^{ - 1} T^{*} + \alpha^{2} \sigma^{2} } \right)$$

$$\left. { + \left\{ { - k^{{*^{2} }} + \left( {4R^{2} } \right)^{ - 1} } \right\}^{ - 2} \left\{ { - T^{*} \sigma^{2} \left( {m_{e} m_{i}^{ - 1} } \right)^{2} - \alpha \sigma^{2} m_{e} m_{i}^{ - 1} } \right\}} \right]\, + \Omega_{{{\text{gi}}}}^{*} M_{i\varphi } \Omega_{{{\text{ge}}}}^{*} M_{e\varphi } \left( { - \Omega_{{{\text{ge}}}}^{*} M_{e\varphi } + 4M_{\varphi } \omega_{z}^{*} } \right)$$

$$+ 2^{ - 1} H_{p}^{2} B_{p} \Omega_{{{\text{gi}}}}^{*} M_{i\varphi } \left( {\Omega_{{{\text{ge}}}}^{*} M_{e\varphi } - 2M_{\varphi } \omega_{z}^{*} } \right) - \Omega_{{{\text{gi}}}}^{*} M_{i\varphi } \left( {4^{ - 1} H_{p}^{2} B_{p} } \right)^{2} - 4\left( {M_{\varphi } \omega_{z}^{*} } \right)^{2} \left( {\Omega_{{{\text{gi}}}}^{*} M_{i\varphi } - 2\Omega_{{{\text{ge}}}}^{*} M_{e\varphi } + 2M_{\varphi } \omega_{z}^{*} } \right)$$

45 $$\left. { + 2\Omega_{{{\text{ge}}}}^{*} M_{e\varphi } M_{\varphi } \omega_{z}^{*} \left( { - \Omega_{{{\text{ge}}}}^{*} M_{e\varphi } + 2^{ - 1} H_{p}^{2} B_{p} } \right) - M_{\varphi } \omega_{z}^{*} 2^{ - 1} H_{p}^{2} B_{p} \left( {4^{ - 1} H_{p}^{2} B_{p} + 4M_{\varphi } \omega_{z}^{*} } \right)} \right]$$

$$\left[ {\left\{ {k^{{*^{2} }} + \left( {4R^{2} } \right)^{ - 1} } \right\}\left\{ { - k^{{*^{2} }} + \left( {4R^{2} } \right)^{ - 1} } \right\}^{ - 1} m_{i} m_{e}^{ - 1} Q} \right]$$

$$= - \left\{ {ik^{*} + \,\left( {2R} \right)^{ - 1} \,} \right\}^{3} \left[ {\left\{ {ik^{*} - \,\left( {2R} \right)^{ - 1} \,} \right\}} \right.\left\{ { - k^{{*^{2} }} + \left( {4R^{2} } \right)^{ - 1} } \right\}^{ - 1} \left[ {m_{i} m_{e}^{ - 1} } \right.\Omega_{{{\text{ge}}}}^{*} M_{e\varphi } \left( {\Omega_{{{\text{gi}}}}^{*} M_{i\varphi } + 2M_{\varphi } \omega_{z}^{*} } \right)$$

$$\left. { - 4M_{\varphi } \omega_{z}^{*} m_{i} m_{e}^{ - 1} \left( {\Omega_{{{\text{gi}}}}^{*} M_{i\varphi } + M_{\varphi } \omega_{z}^{*} } \right) - m_{i} m_{e}^{ - 1} 2^{ - 1} H_{p}^{2} B_{p} M_{\varphi } \omega_{z}^{*} } \right] + \left\{ {ik^{*} - \,\left( {2R} \right)^{ - 1} \,} \right\}^{2} \left[ {\left\{ { - k^{{*^{2} }} + \left( {4R^{2} } \right)^{ - 1} } \right\}^{ - 1} } \right.$$

$$\left. {\left[ {m_{i} m_{e}^{ - 1} \left\{ {\Omega_{{{\text{ge}}}}^{*} M_{e\varphi } - \alpha \left( {\Omega_{{{\text{gi}}}}^{*} M_{i\varphi } + 2M_{\varphi } \omega_{z}^{*} } \right)} \right\} - } \right.m_{i} m_{e}^{ - 1} T^{*} \left( {4^{ - 1} H_{p}^{2} B_{p} + 2M_{\varphi } \omega_{z}^{*} } \right)} \right] + \left\{ { - k^{{*^{2} }} + \left( {4R^{2} } \right)^{ - 1} } \right\}^{ - 2}$$

$$\left. {\left[ { - \sigma m_{i} m_{e}^{ - 1} \left( {\Omega_{{{\text{ge}}}}^{*} M_{e\varphi } - 4^{ - 1} H_{p}^{2} B_{p} - 2M_{\varphi } \omega_{z}^{*} } \right) + \sigma \left( {\Omega_{{{\text{gi}}}}^{*} M_{i\varphi } + 2M_{\varphi } \omega_{z}^{*} } \right)} \right]\,} \right] + \left\{ {ik^{*} - \,\left( {2R} \right)^{ - 1} \,} \right\}^{3} \left[ { - \left\{ { - k^{{*^{2} }} + \left( {4R^{2} } \right)^{ - 1} } \right\}^{ - 1} } \right.$$

46 $$\left. {\left. {\alpha m_{i} m_{e}^{ - 1} T^{*} + \left\{ { - k^{{*^{2} }} + \left( {4R^{2} } \right)^{ - 1} } \right\}^{ - 2} \sigma m_{i} m_{e}^{ - 1} \left( {\alpha + m_{e} m_{i}^{ - 1} } \right)} \right]} \right]$$

$$\left[ {2\left\{ {k^{{*^{2} }} + \left( {4R^{2} } \right)^{ - 1} } \right\}^{2} \left\{ { - k^{{*^{2} }} + \left( {4R^{2} } \right)^{ - 1} } \right\}^{ - 2} \sigma S} \right]$$

$$= - 2\sigma \left\{ {ik^{*} + \,\left( {2R} \right)^{ - 1} \,} \right\}^{3} \left[ { - \left\{ {ik^{*} - \,\left( {2R} \right)^{ - 1} \,} \right\}^{2} } \right.\left\{ { - k^{{*^{2} }} + \left( {4R^{2} } \right)^{ - 1} } \right\}^{ - 2} \left( {\Omega_{{{\text{ge}}}}^{*} M_{e\varphi } - 4^{ - 1} H_{p}^{2} B_{p} - 2M_{\varphi } \omega_{z}^{*} } \right)$$

47 $$\left. { + \left\{ {ik^{*} - \,\left( {2R} \right)^{ - 1} \,} \right\}^{3} \,\left\{ { - k^{{*^{2} }} + \left( {4R^{2} } \right)^{ - 1} } \right\}^{ - 2} \left[ {\alpha - \sigma \left\{ { - k^{{*^{2} }} + \left( {4R^{2} } \right)^{ - 1} } \right\}^{ - 1} m_{e} m_{i}^{ - 1} } \right]} \right],$$

$$\left[ {\left\{ {k^{{*^{2} }} + \left( {4R^{2} } \right)^{ - 1} } \right\}\left\{ { - k^{{*^{2} }} + \left( {4R^{2} } \right)^{ - 1} } \right\}^{ - 1} I} \right]$$

$$= - \left\{ {ik^{*} + \,\left( {2R} \right)^{ - 1} \,} \right\}^{3} \left[ {\left\{ {ik^{*} - \,\left( {2R} \right)^{ - 1} \,} \right\}} \right.\left\{ { - k^{{*^{2} }} + \left( {4R^{2} } \right)^{ - 1} } \right\}^{ - 1} \left[ {\Omega_{{{\text{ge}}}}^{*} M_{e\varphi } \left( { - \Omega_{{{\text{ge}}}}^{*} M_{e\varphi } + 2^{ - 1} H_{p}^{2} B_{p} + 4M_{\varphi } \omega_{z}^{*} } \right)} \right.$$

$$\left. { - 4^{ - 1} H_{p}^{2} B_{p} \left( {4^{ - 1} H_{p}^{2} B_{p} + 4M_{\varphi } \omega_{z}^{*} } \right) - 4\left( {M_{\varphi } \omega_{z}^{*} } \right)^{2} } \right] + \left\{ {ik^{*} - \,\left( {2R} \right)^{ - 1} \,} \right\}^{2} \left[ { - \left\{ { - k^{{*^{2} }} + \left( {4R^{2} } \right)^{ - 1} } \right\}^{ - 1} \left( { - \Omega_{{{\text{ge}}}}^{*} M_{e\varphi } } \right.} \right.$$

$$\left. {\left. { + 4^{ - 1} H_{p}^{2} B_{p} + 2M_{\varphi } \omega_{z}^{*} } \right) + \left\{ { - k^{{*^{2} }} + \left( {4R^{2} } \right)^{ - 1} } \right\}^{ - 2} \left\{ { - 2\sigma \left. {m_{e} m_{i}^{ - 1} \left( {2\Omega_{{{\text{ge}}}}^{*} M_{e\varphi } - 4^{ - 1} H_{p}^{2} B_{p} - 2M_{\varphi } \omega_{z}^{*} } \right.} \right)} \right\}} \right]$$

48 $$\left. { + \left\{ {ik^{*} - \,\left( {2R} \right)^{ - 1} \,} \right\}^{3} \left[ { - \left\{ { - k^{{*^{2} }} + \left( {4R^{2} } \right)^{ - 1} } \right\}^{ - 1} \alpha^{2} + \left\{ { - k^{{*^{2} }} + \left( {4R^{2} } \right)^{ - 1} } \right\}^{ - 2} \left( {2\alpha \sigma m_{e} m_{i}^{ - 1} } \right) - \left\{ { - k^{{*^{2} }} + \left( {4R^{2} } \right)^{ - 1} } \right\}^{ - 3} \left( {\sigma m_{e} m_{i}^{ - 1} } \right)^{2} } \right]} \right]$$

The sextic dispersion relation (Eq. (38)) is transformed into a reduced form in light of the LF approximation with the help of traditional simplification procedure^34^. We are primarily interested in the LF limit because we wish to investigate the cylindrical acoustic waves. In the LF limit $\left( {\Omega^{q} = 0\,,\,\forall \,q > \,1} \right)$, the modified dispersion relation is

49 $$A_{1} \Omega + A_{0} = 0$$

The coefficients $A_{1}$-$A_{0}$ are given in Eqs. (43) and (44), respectively. We then analyze the dispersion relation in four distinct regimes of our interest, namely in quantum (CD) non-planar (cylindrical), quantum planar, classical (CND) non-planar (cylindrical), classical (CND) planar.

### Quantum (CD) non-planar regime

In the quantum non-planar regime, we have the same dispersion relation as given by Eq. (49). Likewise, the coefficients are the same as given by Eqs. (43) and (44).$\alpha$ for the CD case is substituted from Eq. (35).

### Quantum (CD) planar regime

In the quantum planar regime, we have $R \to \infty$. The dispersion relation is the same as Eq. (49). However, the coefficients given by Eqs. (43) and (44) are modified. $\alpha$ for the CD case is substituted from Eq. (35). The cylindrical coordinates are mapped into planar coordinates accordingly. The modified coefficients are given as

$$A_{1} = ik^{{*^{2} }} \eta^{*} \,\left[ {\,\left[ {\left( {ik^{*} } \right)\left[ {\left( {\Omega_{ge}^{*} M_{ey} - 4^{ - 1} H_{p}^{2} B_{p} - 2M_{y} \omega_{z}^{*} } \right)\left[ {2\sigma m_{e} m_{i}^{ - 1} - m_{i} m_{e}^{ - 1} } \right.} \right.} \right.} \right.\left. {\left. { + 2\alpha k^{{*^{2} }} } \right]\,} \right]$$

$$- k^{{*^{2} }} \,\,\left[ {\Omega_{ge}^{*} M_{ey} \left( {\Omega_{ge}^{*} M_{ey} - 2^{ - 1} H_{p}^{2} B_{p} - 4M_{y} \omega_{z}^{*} } \right)} \right.\left. {\left. { + 4^{ - 1} H_{p}^{2} B_{p} \left( {4^{ - 1} H_{p}^{2} B_{p} + 4M_{y} \omega_{z}^{*} } \right) + 4\left( {M_{y} \omega_{z}^{*} } \right)^{2} } \right]\,\,} \right]$$

50 $$- k^{{*^{2} }} \left[ { - 2\alpha \sigma \,m_{e} m_{i}^{ - 1} } \right. + \alpha \,m_{i} m_{e}^{ - 1} \left. {\left. { - k^{{*^{ - 2} }} \left( {\sigma \,m_{e} m_{i}^{ - 1} - m_{i} m_{e}^{ - 1} } \right) - 2\alpha^{2} k^{{*^{2} }} } \right]\,\,} \right]$$

51 $$A_{0} = \left[ { - P - \left( {m_{i} m_{e}^{ - 1} Q} \right)} \right. - \left( {2\sigma S} \right) - \left. {k^{{*^{2} }} \sigma^{2} - I} \right]$$

The different substituted terms in Eq. (51) are modified accordingly.

### Classical (CND) non-planar regime

In the classical non-planar regime, the Bohm potential term is ignored. The dispersion relation is the same as Eq. (49), however, the coefficients $A_{1}$ and $A_{0}$ are modified. $\alpha$ for the classical case is substituted from Eq. (36). The coefficients are modified as

$$A_{1} = - i\left\{ {ik^{*} + \,\left( {2R} \right)^{ - 1} \,} \right\}^{2} \eta^{*} \,\left[ {\,\left[ {\left\{ {ik^{*} - \,\left( {2R} \right)^{ - 1} \,} \right\}\left[ {\left( {\Omega_{ge}^{*} M_{e\varphi } - 2M_{\varphi } \omega_{z}^{*} } \right)\left[ {2\sigma m_{e} m_{i}^{ - 1} - m_{i} m_{e}^{ - 1} } \right.} \right.} \right.} \right.\left. {\left. { - 2\alpha \left\{ { - k^{{*^{2} }} + \left( {4R^{2} } \right)^{ - 1} } \right\}} \right]\,\,} \right]$$

$$\, + \left\{ { - k^{{*^{2} }} + \left( {4R^{2} } \right)^{ - 1} } \right\}\,\,\left[ {\Omega_{ge}^{*} M_{e\varphi } \left( {\Omega_{ge}^{*} M_{e\varphi } - 4M_{\varphi } \omega_{z}^{*} } \right)} \right.\left. {\left. { + 4\left( {M_{\varphi } \omega_{z}^{*} } \right)^{2} } \right]\,\,} \right] + \left\{ {ik^{*} - \,\left( {2R} \right)^{ - 1} \,} \right\}^{2} \left[ { - 2\alpha \sigma \,m_{e} m_{i}^{ - 1} } \right. + \alpha \,m_{i} m_{e}^{ - 1}$$

52 $$\left. {\left. { + \left\{ { - k^{{*^{2} }} + \left( {4R^{2} } \right)^{ - 1} } \right\}^{ - 1} \left( {\sigma \,m_{e} m_{i}^{ - 1} - m_{i} m_{e}^{ - 1} } \right) + 2\alpha^{2} \left\{ { - k^{{*^{2} }} + \left( {4R^{2} } \right)^{ - 1} } \right\}} \right]\,\,} \right]$$

$$A_{0} = \left[ { - P + \left[ {\left\{ {k^{{*^{2} }} + \left( {4R^{2} } \right)^{ - 1} } \right\}\left\{ { - k^{{*^{2} }} + \left( {4R^{2} } \right)^{ - 1} } \right\}^{ - 1} m_{i} m_{e}^{ - 1} Q} \right] + \left[ {2\left\{ {k^{{*^{2} }} + \left( {4R^{2} } \right)^{ - 1} } \right\}^{2} \left\{ { - k^{{*^{2} }} + \left( {4R^{2} } \right)^{ - 1} } \right\}^{ - 2} \sigma S} \right]} \right.$$

53 $$\left. { - \left[ {\left\{ {k^{{*^{2} }} + \left( {4R^{2} } \right)^{ - 1} } \right\}^{3} \left\{ { - k^{{*^{2} }} + \left( {4R^{2} } \right)^{ - 1} } \right\}^{ - 2} \sigma^{2} } \right] + \left[ {\left\{ {k^{{*^{2} }} + \left( {4R^{2} } \right)^{ - 1} } \right\}\left\{ { - k^{{*^{2} }} + \left( {4R^{2} } \right)^{ - 1} } \right\}^{ - 1} I} \right]} \right]$$

The different substituted terms appearing in Eq. (53) are modified as per the approximations stated in “Classical non-planar regime” section.

### Classical (CND) planar regime

In the classical (CND) planar regime, we have $R \to \infty$. Just like the classical non-planar regime, Bohm potential is also ignored herein. The dispersion relation is the same as given by Eq. (49). The coefficients appearing in Eq. (49) are modified as per the considered regime. The cylindrical coordinates are conveniently mapped into planar coordinates. $\alpha$ for the classical case is substituted from Eq. (36). The modified coefficients *A*_*1*_ and *A*_*0*_ are given as

$$A_{1} = ik^{{*^{2} }} \eta^{*} \,\left[ {\,\left[ {\left( {ik^{*} } \right)\left[ {\left( {\Omega_{ge}^{*} M_{ey} - 2M_{y} \omega_{z}^{*} } \right)\left[ {2\sigma m_{e} m_{i}^{ - 1} - m_{i} m_{e}^{ - 1} } \right.} \right.} \right.} \right.\left. {\left. { + 2\alpha k^{{*^{2} }} } \right]\,} \right] - k^{{*^{2} }} \,\,\left[ {\Omega_{ge}^{*} M_{ey} \left( {\Omega_{ge}^{*} M_{ey} - 4M_{y} \omega_{z}^{*} } \right)} \right.\left. {\left. { + 4\left( {M_{y} \omega_{z}^{*} } \right)^{2} } \right]\,\,} \right]$$

54 $$- k^{{*^{2} }} \left[ { - 2\alpha \sigma \,m_{e} m_{i}^{ - 1} } \right. + \alpha \,m_{i} m_{e}^{ - 1} \left. {\left. { - k^{{*^{ - 2} }} \left( {\sigma \,m_{e} m_{i}^{ - 1} - m_{i} m_{e}^{ - 1} } \right) - 2\alpha^{2} k^{{*^{2} }} } \right]\,\,} \right],$$

55 $$A_{0} = \left[ { - P - \left( {m_{i} m_{e}^{ - 1} Q} \right)} \right. - \left( {2\sigma S} \right) - \left. {k^{{*^{2} }} \sigma^{2} - I} \right]$$

The different terms appearing in Eq. (55) are modified as per our approximations (as in “Classical planar regime” section).

The above discussion in the subsections are summarily pointed out as

**Figure 1:**
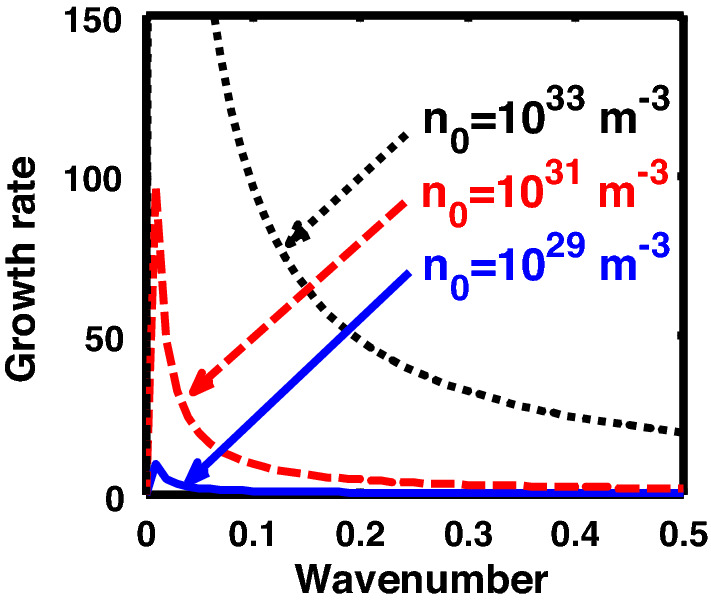
Profile of the normalized growth rate $\left( {\Omega_{i} } \right)$ with variation in the normalized wavenumber $\left( {k^{*} } \right)$. The different lines link to different values of the equilibrium number density $\left( {n_{0} } \right)$ in non-planar (cylindrical) geometry in the quantum regime ($\hbar \ne 0$).

**Figure 2:**
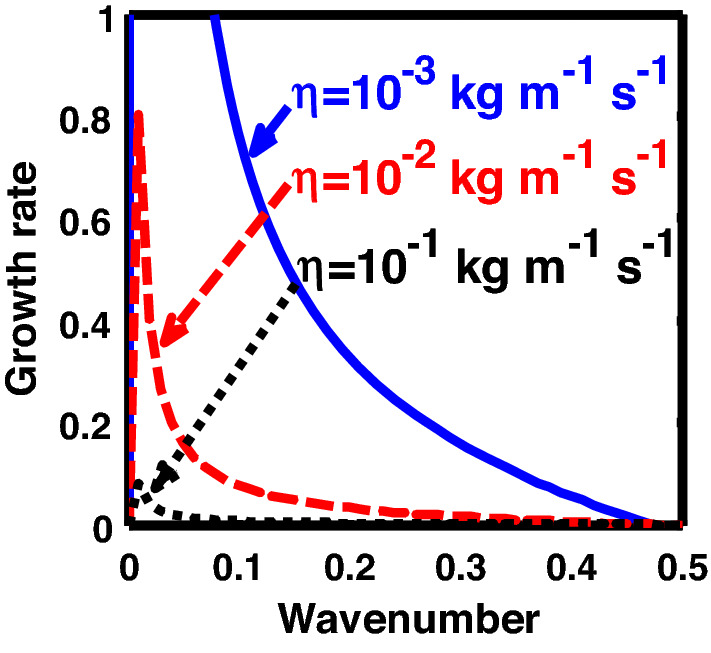
Same as Fig. 1, but for different values of the normalized kinematic viscosity $\left( {\eta^{*} } \right)$.

**Figure 3:**
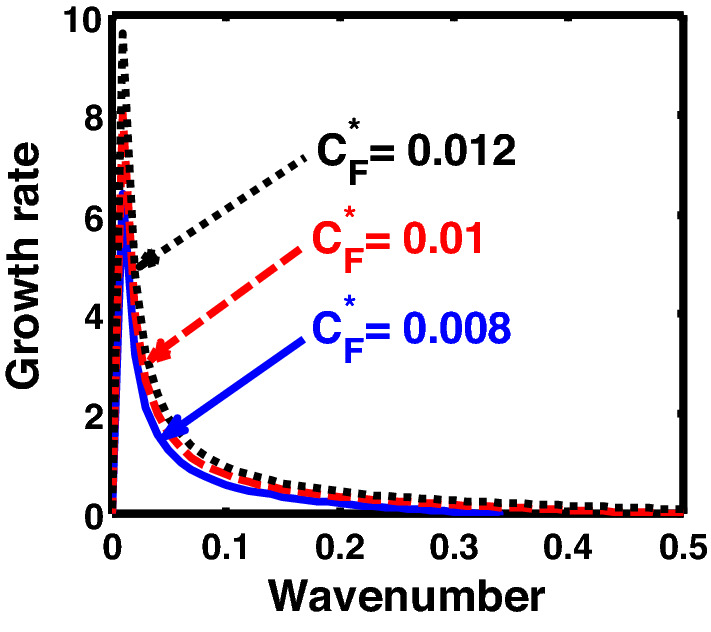
Same as Fig. 1, but for different values of the normalized Coriolis rotational force $\left( {C_{F}^{*} } \right)$.

**Figure 4:**
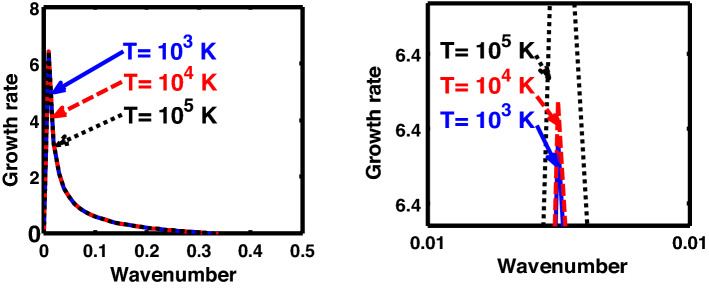
Same as Fig. 1, but for different values of the normalized thermal temperature $\left( {T^{*} } \right)$. The second subplot is the magnified version depicting the peaks (kinks) clearly.

**Figure 5:**
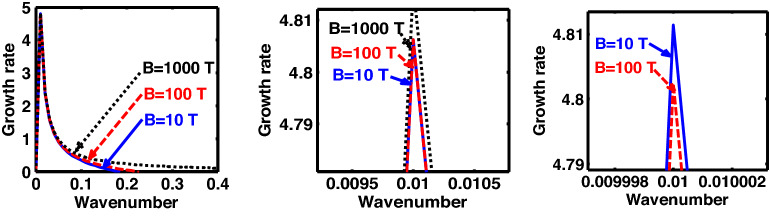
Same as Fig. 1, but for different values of magnetic field $\left( B \right)$. The two subsequent subplots depict the magnified versions clearly highlighting the peaks (kinks).

**Figure 6:**
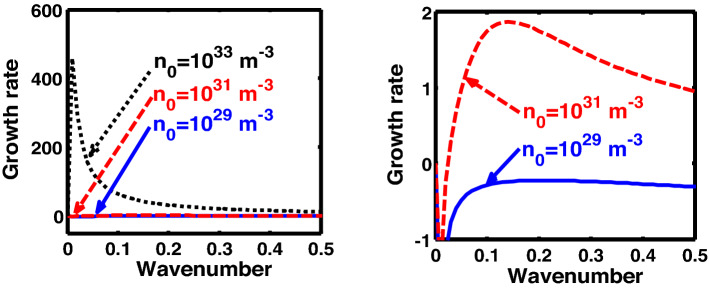
Profile of the normalized growth rate $\left( {\Omega_{i} } \right)$ with variation in the normalized wavenumber $\left( {k^{*} } \right)$. The different lines link to different values of the equilibrium number density $\left( {n_{0} } \right)$ in planar (non-cylindrical) geometry in the quantum regime. The second subplot is the enlarged version highlighting the trends for $n_{0} = 10^{29}$ m^-3^ and $n_{0} = 10^{31}$ m^-3^.

**Figure 7:**
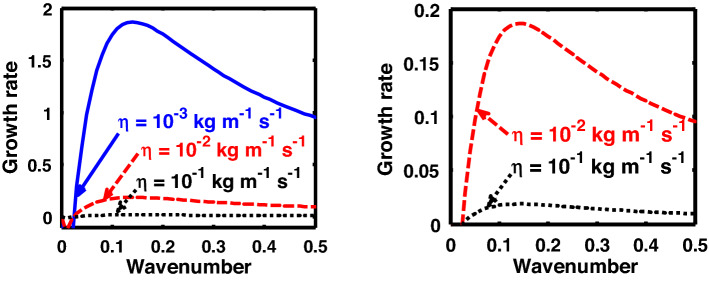
Same as Fig. 6, but for different values of the normalized kinematic viscosity $\left( {\eta^{*} } \right)$. The second subplot is the enlarged version clearly highlighting the trends for $\eta = 10^{ - 2}$ kg m^-1^ s^-1^ and $\eta = 10^{ - 1}$ kg m^-1^ s^-1^.

**Figure 8:**
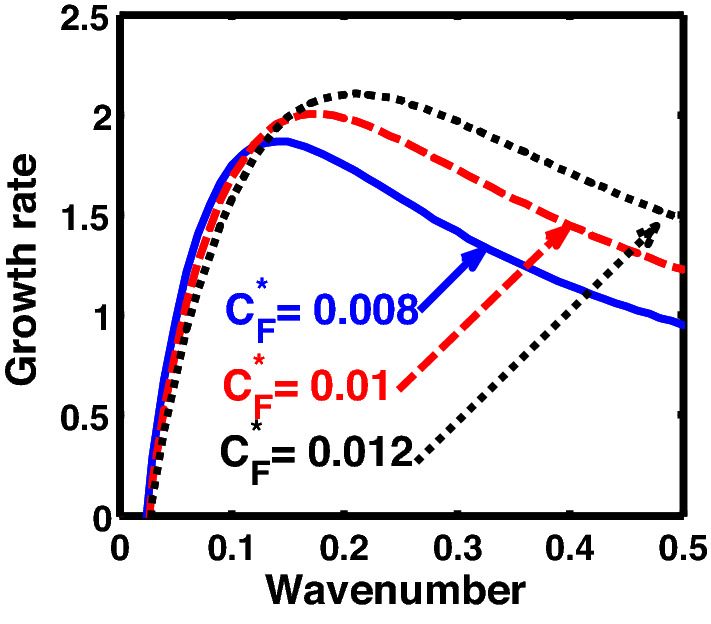
Same as Fig. 6, but for different values of the normalized Coriolis rotational force $\left( {C_{F}^{*} } \right)$.

**Figure 9:**
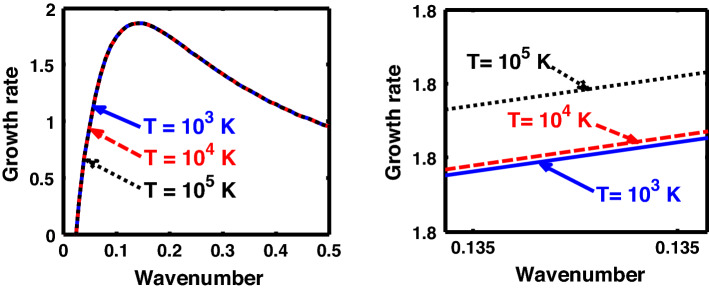
Same as Fig. 6, but for different values of the normalized thermal temperature $\left( {T^{*} } \right)$. The second subplot is the magnified version depicting the peaks clearly.

**Figure 10:**
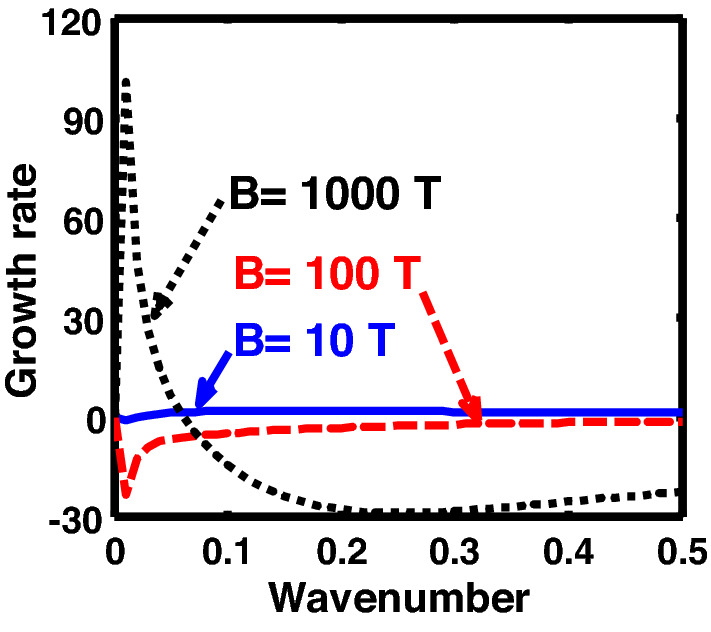
Same as Fig. 6, but for different values of the magnetic field $\left( B \right)$.

**Figure 11:**
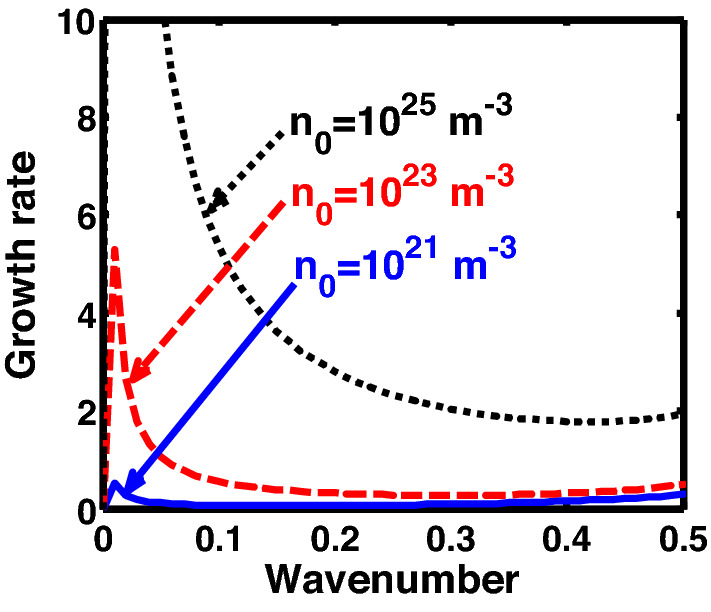
Profile of the normalized growth rate $\left( {\Omega_{i} } \right)$ with variation in the normalized wavenumber $\left( {k^{*} } \right)$. The different lines link to different values of the equilibrium number density $\left( {n_{0} } \right)$ in non-planar (cylindrical) geometry in the classical regime ($\hbar \to 0$).

**Figure 12:**
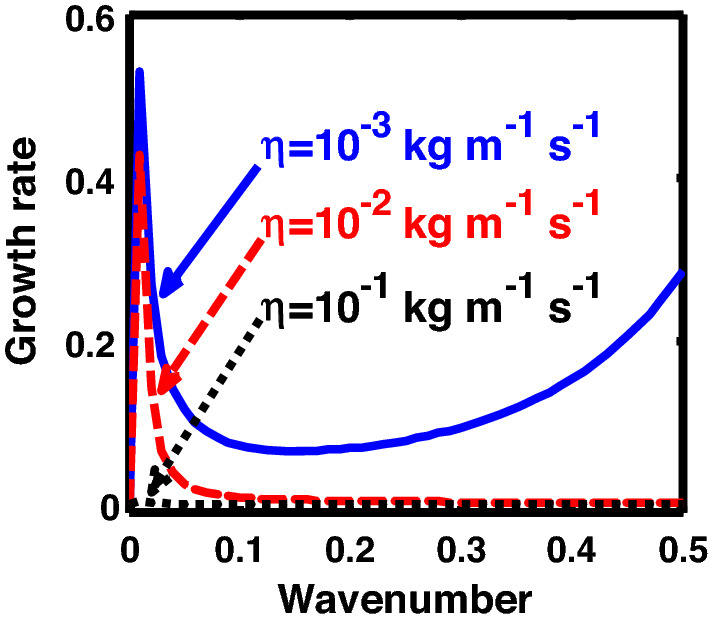
Same as Fig. 11, but for different values of the normalized kinematic viscosity $\left( {\eta^{*} } \right)$.

**Figure 13:**
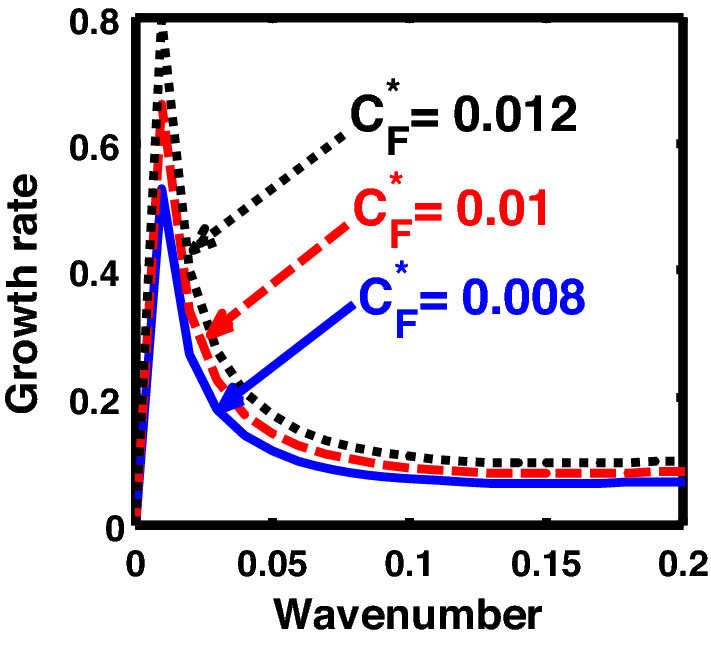
Same as Fig. 11, but for different values of the normalized Coriolis rotational force $\left( {C_{F}^{*} } \right)$.

**Figure 14:**
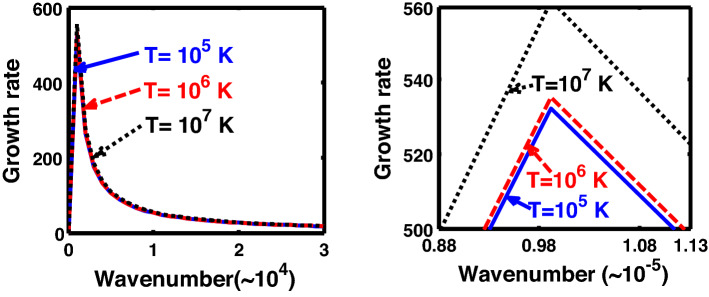
Same as Fig. 11, but for different values of the normalized thermal temperature $\left( {T^{*} } \right)$. The second subplot is the magnified version depicting the peaks (kinks) clearly.

**Figure 15:**
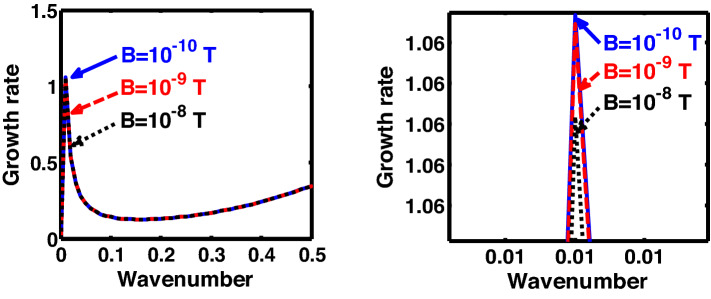
Same as Fig. 11, but for different values of the magnetic field $\left( B \right)$. The second subplot is the magnified version depicting the peaks (kinks) clearly.

**Figure 16:**
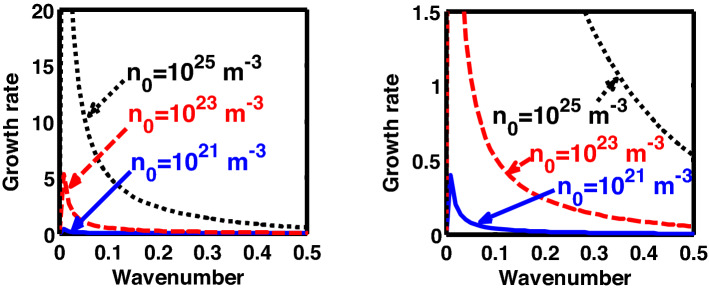
Profile of the normalized growth rate $\left( {\Omega_{i} } \right)$ with variation in the normalized wavenumber $\left( {k^{*} } \right)$. The different lines link to different values of the equilibrium number density $\left( {n_{0} } \right)$ in planar (non-cylindrical) geometry in the classical regime ($\hbar \to 0$). The second subplot is its enlarged version clearly showing the trends for $n_{0} = 10^{21}$ m^-3^ and $n_{0} = 10^{23}$ m^-3^.

**Figure 17:**
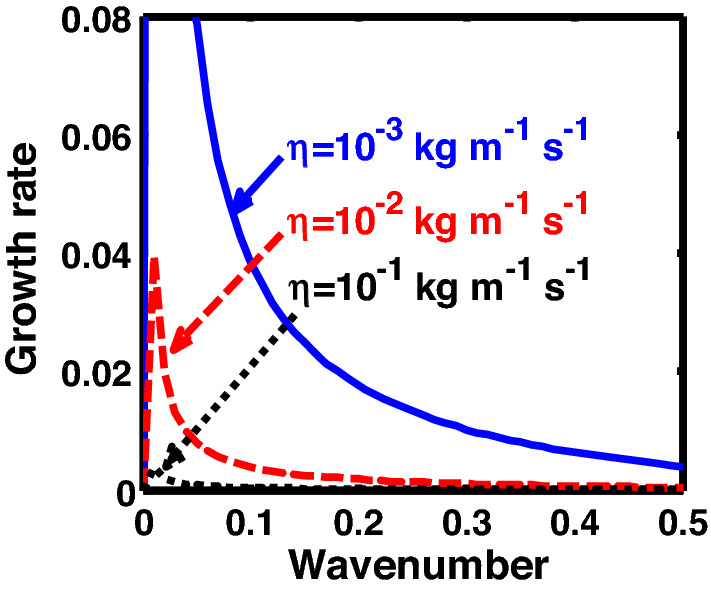
Same as Fig. 16, but for different values of the normalized kinematic viscosity $\left( {\eta^{*} } \right)$.

**Figure 18:**
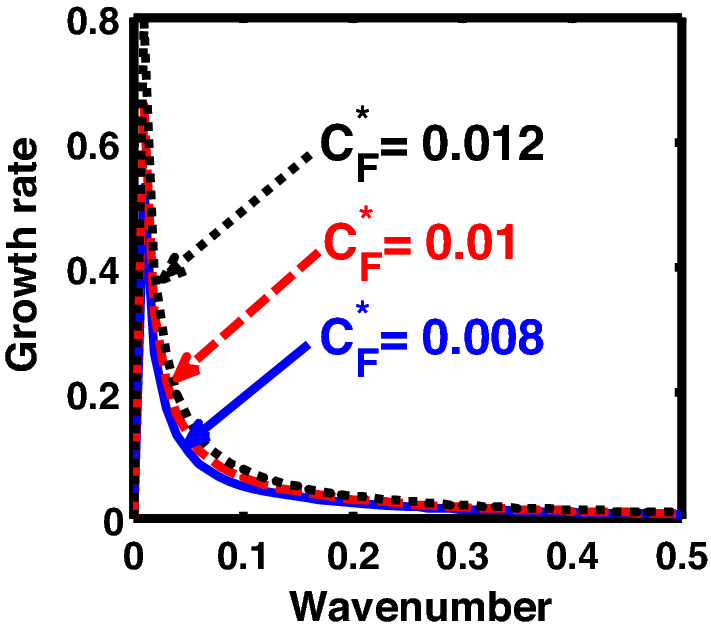
Same as Fig. 16, but for different values of the normalized Coriolis rotational force $\left( {C_{F}^{*} } \right)$.

**Figure 19:**
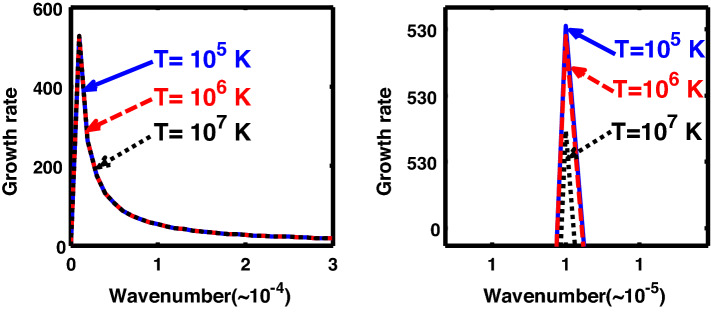
Same as Fig. 16, but for different values of the normalized thermal temperature $\left( {T^{*} } \right)$. The second subplot is the magnified version depicting the peaks (kinks) clearly.

**Figure 20:**
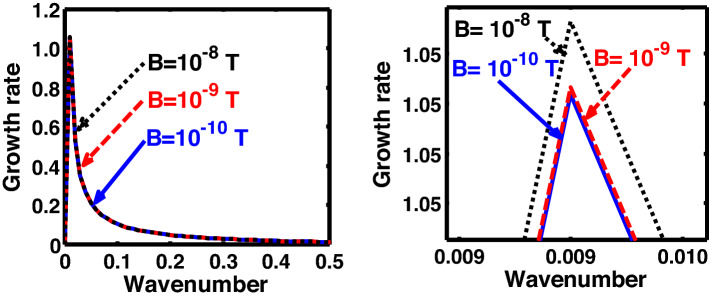
Same as Fig. 16, but for different values of the magnetic field $\left( B \right)$. The second subplot is the magnified version depicting the peaks (kinks) clearly.

- In the quantum non-planar regime, the dispersion relation has the contribution due to the geometric curvature effect, Lorentz force, Coriolis rotational force, kinematic viscosity, quantum parameter, Bohm potential, quantum pressure, temperature, and Jeans-to-plasma oscillation frequency ratio squared. The growth patterns for different parameters are depicted in Figs. 1, 2, 3, 4 and 5.
- In the quantum planar regime, the reduced dispersion relation has the dependencies of all the above terms except the geometric curvature. The growth/damping trends of the same for different relevant parameters are given in Figs. 6, 7, 8, 9 and 10.
- For the classical non-planar regime, the dispersion relation has the dependencies of all the terms as the quantum non-planar regime, except the Bohm potential term. The quantum pressure also gets replaced with the classical pressure. The growth/damp trends for the same are given in Figs. 11, 12, 13, 14 and 15.
- Lastly, for the classical planar regime, the dispersion relation highlights the contribution of all the terms as the classical non-planar regime, except the geometric curvature terms. The growth/damp trends for the relevant parameters in this regime are graphically seen in Figs. 16, 17, 18, 19 and 20.

Thus, it is clearly seen that, in all the four considered distinct regimes, the modified dispersion relation has sensitive dependencies on the multiparametric model coefficients influencing the stability dynamics of the considered plasma system.

## Results and discussions

The excitation and stability features of cylindrical acoustic waves are analyzed by means of a two-component axisymmetric magnetized cylindrical plasma system comprising of electrons and ions. The system is rotating uniformly with its angular velocity directed longitudinally. The electrons evolve under the action of their motion, electrostatic potential, Lorentz force, Coriolis rotational force, Bohm potential and gravitational potential. Meanwhile the ionic dynamics is governed by all of the above mentioned factors, except the Bohm potential term. In addition, kinematic viscosity is retained for the ionic dynamics. The temperature degeneracy of electrons is incorporated via the temperature degeneracy parameter in the equation of state for the electrons. The ions experience the normal classical thermal pressure. A standard cylindrical mode analysis employing the Hankel function yields a generalized linear sextic dispersion relation, which is modified using the LF approximation^24^. A numerical illustrative platform is used to analyze the growth rate corresponding to the acoustic excitation and stability in four parametric windows, namely the quantum non-planar, quantum planar, classical non-planar, and classical planar. Here, the dispersion analysis of current interest in different regimes is systematically carried out by analyzing Eq. (49) graphically, as clearly depicted in Figs. 1, 2, 3, 4, 5, 6, 7, 8, 9, 10, 11, 12, 13, 14, 15, 16, 17, 18, 19, 20. It is noteworthy that different input values used herein exist in the literature^35–42^. There are certain debates regarding the input values and their validity in the classical and quantum domains^43–45^. The number density and temperature range for the quantum regime are given^43^ as 10^24^–10^30^ cm^−3^ and 10^2^–10^7^ K, respectively. In SI units, the number density is 10^30^–10^36^ m^−3^. This is in agreement with the values considered for the quantum regime in the manuscript. Likewise, for the classical regime, the number density and temperature range are given^43^ as 10^6^–10^24^ cm^−3^ and 10^4^–10^7^ K. In SI units, the number density is 10^12^–10^30^ m^−3^. The values for the classical regime in the manuscript are also in good agreement with the specified values of the previous study^43^. The parameters of the study about the non-linear structures in dense magnetoplasmas^44^ is purely for white dwarfs, whereas, the values considered herein are generalized values for quantum and classical regimes. Hence, minor disparities are found between input values of the proposed manuscript and the referred study^44^. The comprehensive review about the fluid description of quantum plasma mostly deals with the pros and cons of the fluid approach of plasma^45^. Apart from a common feature of extreme growth of the fluctuations at extremely large wavelengths, the uncommon features of the same are described and interpreted in the following subsections.

### Quantum (CD) non-planar regime

In Fig. 1, we depict the profile structures of the normalized growth rate $\left( {\Omega_{i} } \right)$ with variation in the normalized wavenumber $\left( {k^{*} } \right)$, which results numerically from Eq. (49), for different values of the equilibrium number density $\left( {n_{0} } \right)$. The different coloured lines link to $n_{0} = 10^{29}$ m^−3^ (blue solid line), $n_{0} = 10^{31}$ m^−3^ (red dashed line), and $n_{0} = 10^{33}$ m^−3^ (black dotted line). As clearly evident from Fig. 1, the growth rate increases with increasing number density. The physical reason behind this can be ascribed to the fact that higher the mass of the system, higher is the possibility of exciting gravitational instability^46^. It couples with the background fluctuations resulting in the growth.

In Fig. 2, we depict the same as Fig. 1, but for different indicated values of the normalized kinematic viscosity $\left( {\eta^{*} } \right)$. The corresponding unnormalized viscosity values ($\eta$) are alongside highlighted for the sake of our easy understanding. The different coloured lines link to $\eta = 10^{ - 3}$ kg m^−1^ s^−1^ ($\eta^{*} = 24.91 \times 10^{ - 6}$, blue solid line), $\eta = 10^{ - 2}$ kg m^-1^ s^−1^ ($\eta^{*} = 24.91 \times 10^{ - 7}$, red dashed line), $\eta = 10^{ - 1}$ kg m^−1^ s^−1^ ($\eta^{*} = 2.49 \times 10^{ - 3}$, black dotted line). The trends shown by the different coloured lines indicate that an increase in the viscosity gradually decreases the instability growth rate, thereby exhibiting stabilizing influence on the system. This can be physically attributed to the fact that with an increase in the viscosity, the cohesion among fluid layers increases^37^. It means that the interspecies force gets enhanced; thereby, restricting the relative fluid motion. As a result, the fluid viscosity here plays a stabilization role against the perturbation dynamics under the current exploration.

In Fig. 3, we indicate the same as Fig. 1, but for different values of the normalized Coriolis rotational force $\left( {C_{F}^{*} } \right)$. The different coloured lines link to $C_{F}^{*} = 0.008$ (blue solid line), $C_{F}^{*} = 0.01$ (red dashed line), and $C_{F}^{*} = 0.012$ (black dotted line). We see that the system has significant growth only in the long-wavelength regime ($k^{*} \to 0$). It is indicated that higher the Coriolis rotational force, higher is the destabilization of the system; and vice-versa. It can be physically attributed to the fact that, higher the Coriolis rotation of the system, higher is the rotational kinetic energy, $E_{r} = \left( {{1 \mathord{\left/ {\vphantom {1 2}} \right. \kern-0pt} 2}} \right)I\omega_{r}^{2} = \left( {{1 \mathord{\left/ {\vphantom {1 2}} \right. \kern-0pt} 2}} \right)MK_{g}^{2} \omega_{r}^{2}$, and vice-versa. Here, *I* is the system moment of inertia around the reference axis of rotation, *M* is the inertial mass of the system with angular velocity $\omega_{r}$ and *K*_*g*_ is its radius of gyration around the same rotation axis. We assume a uniform rotation of the system, which, thereby implicates that $E_{r} \propto M$. It is a well-established fact that heavier objects are gravitationally unstable as compared to their lighter counterparts. Thus, an increase in the Coriolis rotation destabilizes the system, and vice-versa. It is in accordance with the previous results by us^20^ and astronomical evidences observed by others^47,48^.

As in Fig. 4, we depict the same as Fig. 1, but for different indicated values of the normalized thermal temperature $\left( {T^{*} } \right)$. Here, just like Fig. 2, the unnormalized values of the temperature are indicated in Fig. 4. The different coloured lines link to $T = 10^{3}$ K ($T^{*} = 8.26 \times 10^{ - 8}$, blue solid line), $T = 10^{4}$ K ($T^{*} = 8.26 \times 10^{ - 7}$, red dashed line), and $T = 10^{5}$ K ($T^{*} = 8.26 \times 10^{ - 6}$, black dotted line). The different coloured lines clearly indicate that, an increase in the temperature destabilizes the system, and vice-versa. It is indeed a well-established fact that a temperature increase enhances the system kinetic energy, and so on. It, hereby, randomizes the system at the cost of enhanced particle thermal motion resulting in destabilization of the system. In other words, it is noteworthy that microscopic thermal motions of the individual constitutive particles significantly contribute to the bulk development of an anti-centric thermal pressure force (outward, randomizing) against the concentric gravitational counterpart (inward, organizing), causing the bulk destabilization consequences.

In a similar way, Fig. 5 shows the same as Fig. 1, but for different values of the magnetic field. The different coloured lines correspond to $B = 10$ T (blue solid line), $B = 100$ T (red dashed line), and $B = 1000$ T (black dotted line). An interesting hybrid trend of growth peaks in different magnetic conditions is found to exist. It is against the previous cases showing a definite multiparametric increasing or decreasing growth pattern (Figs. 1, 2, 3, 4). In other words, Fig. 5 shows a unique admixture of fluctuation growth patterns. Here, the growth rate is highest for $B = 1000$ T, followed by the subsequent gradually weaker growths produced at $B = 10$ T and $B = 100$ T, respectively. The non-uniformity in the instability growth-peak order with the magnetic field strength found here is a new and unique behaviour exhibited by this categorical class of collective fluctuation dynamics.

### Quantum (CD) planar regime

In Fig. 6, we depict the same as Fig. 1, but for the quantum planar geometric regime. The colour spectral coding is the same as that of Fig. 1. Clearly, the growth rate increases with increasing number density, and vice-versa. This is physically due to the well-established fact that heavier objects are gravitationally more unstable as compared to their lighter counterparts on the astrophysical scales. Even though the trend shown by Fig. 1 (quantum non-planar regime) is the same as Fig. 6 (quantum planar regime), the growth rate of the considered fluctuation dynamics is considerably higher for the latter.

In an analogous way, Fig. 7 shows the same as Fig. 2, but for the quantum planar regime. The colour coding used here is the same as that of Fig. 2. Clearly, the growth rate decreases with increase in the kinematic viscosity, and vice-versa. It may, therefore, be inferred that an enhancement in the viscosity leads to the stabilization of the self-gravitating system, and vice-versa. The physical reason behind this is the same as described in Fig. 2. Viscosity playing as a stabilizing role in self-gravitating systems is a well-known fact established in the literature^37^.

Similarly, Fig. 8 shows the same as Fig. 3, but for the quantum planar regime. As can be clearly seen herefrom, the growth rate of the system increases with an increase in strength of the Coriolis rotational force. The physical reason behind this behaviour is the same as that of Fig. 3.

Figure 9 depicts the same as Fig. 4, but for the quantum planar regime. An enhancement in the temperature increases the kinetic energy of the constitutive particles, thereby increasing the disturbance in the system. As a result, the instability growth rate of the considered instability increases with the temperature, and vice-versa.

Figure 10 shows the same as Fig. 5, but for the quantum planar regime. The colour spectral coding used here is exactly the same as that used in Fig. 5. An absurd behaviour is seen to exist in the case of the magnetic field enhancement followed by a simultaneous existence of both growth dips and peaks. More specifically, while $B = 10$ T and $B = 100$ T give growth rate dips; in contrast, $B = 1000$ T results in a growth peak, and so forth.

### Classical (CND) non-planar regime

In Fig. 11, we depict the profile structures of the normalized growth rate $\left( {\Omega_{i} } \right)$ with variation in the normalized wavenumber $\left( {k^{*} } \right)$ for different values of the equilibrium number density $\left( {n_{0} } \right)$. The different coloured lines link to $n_{0} = 10^{21}$ m^−3^ (blue solid line), $n_{0} = 10^{23}$ m^−3^ (red dashed line), and $n_{0} = 10^{25}$ m^−3^ (black dotted line). It is found that an enhancement in the equilibrium number density increases the growth rate, and vice-versa. This growth behaviour is the same as that observed in both the quantum regimes discussed previously (Figs. 1, 6). The physical insight behind such instability growth features is the same as that already described in Fig. 1.

Figure 12 shows the same as Fig. 11, but for different values of the normalized kinematic viscosity $\left( {\eta^{*} } \right)$. Here, the unnormalized (normalized) values of the kinematic viscosity are indicated for our easy comprehension. The different coloured lines link to $\eta = 10^{ - 3}$ kg m^−1^ s^−1^ ($\eta^{*} = 3.03 \times 10^{4}$, blue solid line), $\eta = 10^{ - 2}$ kg m^−1^ s^−1^ ($\eta^{*} = 3.03 \times 10^{5}$, red dashed line), $\eta = 10^{ - 1}$ kg m^−1^ s^−1^ ($\eta^{*} = 3.03 \times 10^{6}$, black dotted line). An enhancement in the value of kinematic viscosity again stabilizes the system. The physical reason behind this trend is the same as in Fig. 2.

Figure 13 shows the same as Fig. 11, but for different values of the Coriolis rotational force $\left( {C_{F}^{*} } \right)$. The different coloured lines correspond to $C_{F}^{*} = 0.008$ (blue solid line), $C_{F}^{*} = 0.01$ (red dashed line), and $C_{F}^{*} = 0.012$(black dotted line). It clearly indicates that the Coriolis force enhancement destabilizes the system, and vice-versa. The physical reason is the same as Fig. 3.

Figure 14 shows the same as Fig. 11, but for different values of the normalized thermal temperature. The different coloured lines link to $T = 10^{5}$ K ($T = 1.84 \times 10^{3}$, blue solid line), $T = 10^{6}$ K ($T = 1.84 \times 10^{4}$, red dashed line), and $T = 10^{7}$ K ($T = 1.84 \times 10^{5}$, black dotted line). It can be clearly seen that the growth rate of the system increases with an increase in the temperature in the considered configuration, and so forth. It hereby implies that the temperature acts as a destabilization agent under the joint action of all the considered factors.

Figure 15 shows the same as Fig. 11, but for different values of the magnetic field. The different coloured lines correspond to $B = 10^{ - 10}$ T (blue solid line), $B = 10^{ - 9}$ T (red dashed line), and $B = 10^{ - 8}$ T (black dotted line). In contrast to the hybrid behaviour displayed in both the quantum regimes, the magnetic field, in case of classical non-planar regime, shows a definite trend. The growth rate of the instability decreases on increasing the magnetic field, and vice-versa. It is founded on the basics of plasma confinement processes in an external magnetic field. Due to an increase in plasma confinement on the magnetic field enhancement, the instability growth rate of the system decreases, and vice-versa. The same has also been pointed out in the previous investigations reported in the literature elsewhere^20^.

### Classical (CND) planar regime

In the classical (CND) regime, Fig. 16 shows the same as Fig. 11, but for the plane geometry approximation. It can be clearly seen herein that the growth rate increases with the equilibrium number density, and vice-versa. The explanation behind the observed trend is the same as Fig. 1.

Again, Fig. 17 shows the same as Fig. 12, but for the classical planar regime. As clearly evident herein, the growth rate decreases with increase in the kinematic viscosity value, and vice-versa. The explanation behind this growth trend is already presented in case of Fig. 2.

Furthermore, Fig. 18 shows the same as Fig. 13, but for the classical plane-geometry regime. It can be clearly inferred from here that the growth rate increases with the strength of the Coriolis rotational force, and vice-versa. The physical mechanism operating behind this growth pattern trend is the same as Fig. 3; and so forth.

Likewise, Fig. 19 shows the same as Fig. 14, but for the classical planar regime. Interestingly, Fig. 19 shows an opposite growth trend against Fig. 14. In other words, in Fig. 19, a temperature enhancement stabilizes the system, and vice-versa. That is, the growth rate of the system decreases with an increase in the temperature, and vice-versa. It is a well-known fact that an increase in the temperature increases the kinetic energy of the system, and so forth. Thus, an excessive kinetic energy gained on a high temperature scale is dissipated away to the surroundings, thereby reducing the kinetic energy of the system. As a consequence, higher the temperature, higher is the kinetic energy, and higher will be the rate of dissipation, thereby decreasing the instability growth rate under consideration.

At the last, Fig. 20 shows the same as Fig. 15, but for the classical planar regime. Interestingly, an opposite behavioural pattern of the instability growth is observed herein against Fig. 15. That is, the growth rate of the instability increases with the strength of the magnetic field, and vice-versa. This is because, for the plasma to be confined in a magnetic field, a certain curvature drift effect is required, which is, however, missing in the case of the classical planar regime^49^. Moreover, enhanced magnetic field strength increases the gyrofrequency of the constitutive particles. It hereby leads to the system destabilization on the Larmor kinetic footing.

On the basis of the above described results, it can clearly be inferred that, in the quantum regime, the equilibrium number density plays the most dominant role in destabilizing the system. However, in the classical regime, the system temperature plays a major role in stabilizing/destabilizing the system. Moreover, the destabilizing nature of rotational force is observationally accounted in many white dwarf stars^47^ and circumstellar discs^48^. To sum up, a compact table outlining a concise contrast on the fluctuation dynamics in all the four distinct considered regimes for the sake of readers can be given in Table 1 as follows.

**Table 1:** Fluctuation dynamics in different regimes.

S. No	Parameter	Quantum non-planar	Quantum planar	Classical non-planar	Classical planar
1	Equilibrium number density	Destabilizer	Destabilizer	Destabilizer	Destabilizer
2	Kinematic viscosity	Stabilizer	Stabilizer	Stabilizer	Stabilizer
3	Coriolis rotation	Destabilizer	Destabilizer	Destabilizer	Destabilizer
4	Temperature	Destabilizer	Destabilizer	Destabilizer	Stabilizer
5	Magnetic field	Mixed role	Absurd	Stabilizer	Destabilizer

## Conclusions

In our proposed semi-analytic study, a two-component quantum hydrodynamic plasma model is presented to analyze the excitation and stability dynamics of cylindrical acoustic waves excitable in magnetized cylindrical astrophysical structures. The considered plasma system is gyrogravitating in nature. The electrons evolve under the conjoint action of electrostatic potential, Lorentz force, Coriolis rotational force, Bohm potential, and temperature degeneracy pressure effects. The temperature degeneracy parameter is incorporated in the electronic dynamics by means of the electronic equation of state. The temperature degeneracy parameter in the equation of state results in a CD quantum (Fermi) pressure and a CND classical (thermal) pressure in judicious approximations in correlation with realistic scenarios. The constitutive ionic fluid dynamics is modelled jointly with the electrostatic potential, Lorentz force, Coriolis rotational force, and kinematic viscosity. Thus, the electronic fluid is affected by the quantum potential, whereas the ionic fluid by the kinematic viscosity, in contrast, in isolation. The ions are acted upon by the classical thermal pressure. A cylindrical wave analysis employing the Hankel function yields a linear generalized sextic dispersion relation. The LF acoustic regime is then thoroughly investigated in four distinct parametric windows of practical importance. It includes the quantum non-planar, quantum planar, classical non-planar, and classical planar. The obtained results on the diverse stability factors in an itemized form are summarily given as follows.

A) In the quantum non-planar regime, the equilibrium number density, Coriolis rotational force, and temperature destabilize the system. The viscous influence stabilizes the system. The magnetic field shows a mixed behaviour in the instability dynamics.
B) In the quantum planar regime, the behaviour of the equilibrium number density, Coriolis rotational force, temperature, and viscosity remain the same as in the quantum non-planar regime. However, the magnetic field shows absurd peaks and dips in the instability dynamics in this quantum planar regime only.
C) In the classical non-planar regime, the equilibrium number density, temperature, and Coriolis rotational force destabilize the system. The viscosity and magnetic field are found to stabilize the astrofluid system under consideration.
D) In the classical planar regime, the magnetic field shows the opposite behaviour to that in the classical non-planar regime. It aids in destabilizing the system, along with other factors, like the equilibrium number density, temperature, and Coriolis rotational force. The fluid viscosity, here too, is found to stabilize the considered cylindrical fluid system in accordance with the hydrodynamical first principle.

The physical parameters based on which the dispersion relation of the proposed cylindrical model are analyzed are the number density, viscosity, rotation, temperature, and magnetic field. The proposed theoretic analysis can be extensively applied to study diverse cylindrical waves excitable in elongated molecular clouds, filamentary structures, magnetized arms of spiral galaxies, and so on^28–30^. It has been seen that circumstellar discs undergo viscous evolution^50^. Circumstellar discs with masses more than 10% of the central star are more susceptible to gravitational instability^46^ (more number density, more mass). The mass may also increase by means of mass accretion due to rotational processes^46,48^. Magnetic field also plays a significant role in the evolution of the protoplanetary disks^51^. The chemistry of the disc and the evolution of the grain population are affected by magnetically driven mixing^51^. The direction of migration of planets is determined by the effective viscous reaction of the disc^51^. As a result, it can be seen that the presented analysis has an extensive reliability and validity.

It is finally admitted that, like many other theoretical model analyses, our model is not completely free from formalism limitations. Approximate input values, although judiciously used herein for certain rotation parameters, might perhaps have slightly affected the accuracy of the obtained results. Also, the consideration of non-linearity and differential rotation would actually improve the realistic applicability of the results. There exists no sufficiency of actual astronomical stability data needed for a complete validation and concrete reliability checkup of our proposed theoretic investigation. Against this backdrop, a refined model development with the aforesaid key factors taken into full consideration is left here now for a future course of integrated study on astrophysical cylindrical stability analyses in diverse circumstances.

## Data Availability

All data generated or analyzed during this study are included in this published article.
